# The CATH Hierarchy Revisited—Structural Divergence in Domain Superfamilies and the Continuity of Fold Space

**DOI:** 10.1016/j.str.2009.06.015

**Published:** 2009-08-12

**Authors:** Alison Cuff, Oliver C. Redfern, Lesley Greene, Ian Sillitoe, Tony Lewis, Mark Dibley, Adam Reid, Frances Pearl, Tim Dallman, Annabel Todd, Richard Garratt, Janet Thornton, Christine Orengo

**Affiliations:** 1 Institute of Structural and Molecular Biology, University College London, London WC1E 6BT, UK; 2 European Bioinformatics Institute, Wellcome Trust Genome Campus, Hinxton, Cambridge CB10 1SD, UK; 3 Institute de Fisica de Sao Carlos, Universidade de Sao Paulo, Caixa Postal 369, Sao Carlos, SP, Brazil

**Keywords:** PROTEINS

## Abstract

This paper explores the structural continuum in CATH and the extent to which superfamilies adopt distinct folds. Although most superfamilies are structurally conserved, in some of the most highly populated superfamilies (4% of all superfamilies) there is considerable structural divergence. While relatives share a similar fold in the evolutionary conserved core, diverse elaborations to this core can result in significant differences in the global structures. Applying similar protocols to examine the extent to which structural overlaps occur between different fold groups, it appears this effect is confined to just a few architectures and is largely due to small, recurring super-secondary motifs (e.g., αβ-motifs, α-hairpins). Although 24% of superfamilies overlap with superfamilies having different folds, only 14% of nonredundant structures in CATH are involved in overlaps. Nevertheless, the existence of these overlaps suggests that, in some regions of structure space, the fold universe should be seen as more continuous.

## Introduction

The initial expansion of the protein databank (PDB) in the mid-1990s inspired the creation of several hierarchical (SCOP, Murzin et al., 1995; CATH, Orengo et al., 1997; and 3Dee, Siddiqui et al., 2001 and Dengler et al., 2001) and nonhierarchical (HOMSTRAD, Mizuguchi et al., 1998) protein domain classifications. In CATH, structures are first divided into their constituent domains and then classified at four major levels: (C)lass, (A)rchitecture, (T)opology or fold, and (H)omologous superfamily. SCOP, another comprehensive classification, employs similar divisions; however, architectures, which describe the overall shape of the folds, are not explicitly recognized. Since these resources were established, there has been an exponential expansion in the number of solved structures, revealing a rich diversity of protein folds and evolutionary relationships.

The first detailed analysis of CATH, published in 1997, was based on ∼8,000 domains classified from the PDB at that time. CATH version 3.1 has expanded by over 10 fold to 93,885 domains, with twice the number of fold groups (1100 from 505) (see Figure S1 available online). This significant increase is due to advances in structure determination, in addition to the structural genomics initiatives (Todd et al., 2005; Marsden et al., 2006) targeting a greater proportion of novel and highly divergent folds than traditional structural biology. Furthermore, there have been increases in the sensitivity of methods used for detecting structural similarities (Redfern et al., 2007; Kolodny et al., 2005; Panchenko and Madej, 2005) and for recognizing very remote homologs from sequence (Sadreyev and Grishin, 2003; Reid et al., 2007), providing greater opportunity to detect and analyze fold similarities and distant evolutionary relationships.

One key feature of the structural universe identified by our original analysis was the recurrence of common motifs (e.g., α-hairpin motifs) that cause overlaps in fold space as the result of a phenomenon called the “Russian doll effect” (Orengo et al., 1997). This phrase described how, by successively adding small structural motifs, it was possible to walk from one fold to another, and it was subsequently commented on by others (Krishna and Grishin, 2005; Friedberg and Godzik, 2005; Sippl et al., 2008). Another extensive analysis of CATH also identified overlapping structural motifs both within and between different CATH architectures (Harrison et al., 2002).

More recently, a detailed look at the domains within larger CATH superfamilies has revealed the extent to which the structures adopted by different homologs can be extended or embellished in different ways, beyond the conserved structural core. Indeed, in some superfamilies there is significant structural variation whereby some relatives contain three times more secondary structure elements than others (Reeves et al., 2006). Although the folding arrangement in the common structural core tends to be conserved, if the global structure is considered, evolutionary divergence appears to effect a transition from one fold to another. Furthermore, this variation is unlikely to be due to errors in the classification as the domains are only grouped in the same superfamily where there are clear multiple lines of evidence (significant sequence profile scores, functional similarity, and structural similarity).

Several evolutionary mechanisms (residue mutations, insertions, deletions, and circular permutations) underlie this structural variability and can result in the insertion and rearrangements of secondary structure elements (Grishin, 2001; Lupas et al., 2001). For hierarchical structure classifications, such as CATH and SCOP, one of the greatest challenges is in determining at what point a variation (e.g., the insertion of several secondary structures or a large structural motif) presents a new fold. For this reason, there has been much speculation in the literature as to whether the structural universe is better viewed as a continuum (McGuffin et al., 2001; Harrison et al., 2003; Grant et al., 2004; Kolodny et al., 2006).

Despite the controversies around structural classifications, structure-based domain classifications have been shown to be valuable for deriving phylogenetic trees (Yang et al., 2005) and revealing evolutionary mechanisms in different kingdoms of life (Wilson et al., 2006; Ranea et al., 2007).

Another advantage of organizing domain structures into superfamilies and fold groups has been the detection of bias in the populations of these classification levels. An analysis of CATH revealed the existence of ten superfolds, which were overrepresented in the PDB (Orengo et al., 1994) and appeared to comprise multiple superfamilies, unlike the majority of other fold groups. Similar observations were made using the SCOP classification (Hubbard et al., 1998), and more recent analysis of completed genomes has shown that there is a genuine bias toward these folds in the genomes (Lee et al., 2005; Orengo and Thornton, 2005).

With the significant increase in the number and population of fold groups in CATH over the last fifteen years, we have decided to revisit the distribution of structures across the classification and to examine whether the structural divergence in some superfamilies and overlap between fold groups is posing a serious challenge to the concept of a hierarchical classification scheme.

Our analysis has revealed that, although the number of domain structures in CATH has increased by over 10 fold, the numbers of architectures and core folds have grown more slowly. Furthermore, many of the fold groups shown to dominate the classification 15 years ago are still among the most highly populated. We show that for a small percentage of superfamilies (4%), significant structural divergence is observed among relatives. If a threshold of normalized RMSD <5Å (Redfern et al., 2007), is used to cluster structurally similar groups (SSGs) of domains, these superfamilies account for nearly 25% of distinct SSGs in CATH. They are also highly populated, accounting for 40% of predicted domain structures in the sequences of completed genomes.

Although most superfamilies do not structurally overlap with other fold groups in CATH, there are clearly overlaps between superfamilies in different folds in a small subset of architectures. These overlaps tend to occur between small domains, comprising fewer than six secondary structures, and are largely associated with super-secondary motifs (eg α-hairpins, αβ-motifs, and β-meanders), which recur or account for a large proportion of the fold. Although 24% of superfamilies are involved in overlaps, only 32% of nonredundant structures within them overlap with different folds (14% of all nonredundant structures in CATH). However, the presence of these overlaps suggests that for these architectures, fold space should be viewed as more continuous in nature.

Although extreme structural divergence in superfamilies and structural overlaps between fold groups potentially challenge the notion of a hierarchical classification in CATH, we present strategies for coping with these phenomena. The T-level in CATH will group superfamilies sharing a common topology or folding arrangement in the evolutionary conserved cores of their domains. These similarities will be identified by manual inspection guided by automated structure comparison and analysis tools. In addition, CATH will also formally identify structural links between domains in different superfamilies to capture the more continuous nature of the relationships that exist in some regions of the structural universe.

## Results and Discussion

### Population of the CATH Hierarchy

There are 93,885 domains in version 3.1 of CATH, a 10-fold increase since the last detailed analysis of CATH in 1997 (Orengo et al., 1997). Despite this considerable expansion, Figure S1 shows that the number of superfamilies, folds, and architectures has increased much more slowly. Figure S2 shows a representative from each architecture in CATH, whereas the Protein Chart (Figure S3) shows representatives from CATH domain folds of increasing number of secondary structures for each regular architecture.

In CATH, the fold level is manually assigned, guided by automatic structure comparison. If a newly solved domain structure does not superpose on any classified domain in CATH with a normalized RMSD <5Å (see Experimental Procedures) and exhibits a previously unseen topological arrangement of secondary structures in the core, then it is classified as having a novel fold. According to this definition, only about 1% of nonidentical structures solved by conventional structural biology in 2004 were found to adopt novel folds (3% for structural genomics; see Figure S4).

As first noted in 1994 (Orengo et al., 1994) and supported by subsequent analyses (Orengo et al., 1997; Chandonia and Brenner, 2005), there is still a bias in the population of fold groups and superfamilies, with the majority being quite small (Figure S5). The top 20 most highly populated fold groups in CATH (in terms of sequences in Gene3D) account for 46% of nonredundant domain sequences that belong to CATH superfamilies in the genomes.

### Analysis of the Structural Drift and Variation within CATH Domain Superfamilies

CATH classifies all structures that have diverged from a common ancestor into superfamilies (see Supplemental Data, section 8). We analyzed the extent to which superfamilies diverge structurally by superposing relatives and clustering those with similar structures (see Experimental Procedures). Relatives that can be superposed with a normalized RMSD <5Å (see Experimental Procedures) were clustered into the same SSG. This threshold was chosen because it was the value that distinguished best between homologous and analogous domains in the same fold group. That is, the majority of homologous domains superpose with normalized RMSD <5Å, whereas the majority of analogous domains in the same fold group superpose above this value (see Figure S6). Structural divergence across a superfamily can then be assessed in a simple manner by considering the number of SSGs it contains (for full details, see Experimental Procedures).

**Figure 1:**
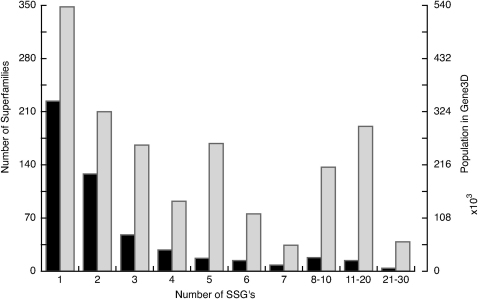
Relationship Between the Degree of Structural Diversity and Population of the Superfamilies in the Genomes Structural diversity was measured by the number of SSGs, shown as black bars (see Experimental Procedures). Gray bars indicate number of sequences.

It can be seen from Figure 1 that, although many superfamilies comprise only one or two SSGs, some superfamilies comprise many more, suggesting that there is considerable structural drift across the superfamily. Although protein structure is more highly conserved than sequences through evolution (Chothia and Lesk, 1986), our original analysis of CATH in 1997 revealed the surprising extent to which some relatives could diverge in structure. Subsequent studies using other approaches have revealed that this phenomenon is especially pronounced in some superfamilies, where relatives vary in size by three fold or more, usually as a result of extensive secondary structure insertions that embellish the conserved structural core of the superfamily (Reeves et al., 2006).

Figure 1 also shows that a very small number of structurally diverse superfamilies (containing between 11 and 20 SSGs) account for a disproportionate number of domain sequences in the genomes. These superfamilies are members of the superfolds, which also account for a large number of structures in the PDB.

**Figure 2:**
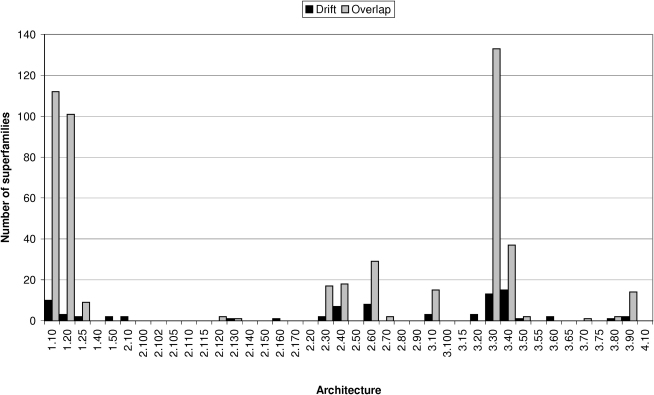
Plot Showing the Number of Structurally Diverse Superfamilies and Overlapping Superfamilies in Each Architecture Structurally diverse superfamilies (shown in black) are defined as those superfamilies with 5 or more SSGs. Overlapping superfamilies are shown in gray. The architectures with the highest proportion of structurally diverse superfamilies are 3.40 (3 layer (αβα) sandwich), 3.30 (2 layer (αβ) sandwich), 2.60 (2 layer (ββ) sandwich), 1.10 (orthogonal bundle), and 2.40 (β barrel). The most overlapping architectures are 3.30 (2 layer (αβ) sandwich), 1.10 (orthogonal bundle), 1.20 (up-down bundle), 3.40 (40 (3 layer (αβα) sandwich), 2.60 (2 layer (ββ) sandwich), 2.40 (β barrel), and 2.30 (β roll). See Results for more details.

If we define highly structurally diverse superfamilies as those comprising five or more SSGs, it can be seen that the two and three layered β and αβ-architectures contain a disproportionately higher number of highly diverse superfamilies than other architectures (see Figure 2). Furthermore, nearly half of the nonredundant structures within these architectures adopt one of four superfolds (Rossmann, 3.40.50; αβ-plait, 3.30.70; TIM barrels, 3.20.20; and immunoglobulin, 2.40.60).

**Figure 3:**
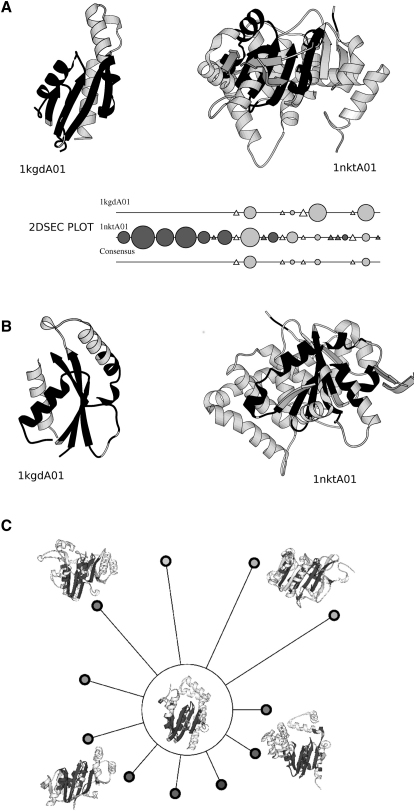
Structural Diversity of Two P-Loop Nucleotide Hydrolase Domains (A) Molscript pictures of the two P loop nucleotide hydrolase domains guanylate kinase (1kgdA01) and translocation atpase (1nktA01). Black indicates structural regions common to both domains, and gray indicates structural regions specific to a domain. The corresponding 2DSEC plot shows secondary structures (circle, α-helix; triangle, β strand) common to both domains (light gray) and specific secondary structures for a domain (dark gray). The size of the symbol reflects the number of residues in the secondary structure element. Following a superposition of these two domains, the “Consensus” plot highlights secondary structures common to both domains. The normalized RMSD calculated following the superposition of these domains is 14.5 Å. (B) Edge on view of the two domains shown in (A). (C) Foldspin plot showing structural diversity exhibited by selected relatives from the P loop hydolase superfamily (3.40.50.300). The “common structural core” between the central structure and other domains in the superfamily is shown in dark gray. The length of the spokes reflects the normalized RMSD measured for a particular relative superposed onto the central domain. Protein structure figures created using Molscript (Kraulis, 1991).

Among the superfamilies exhibiting extreme structural drift, there are four Rossmann fold (3.40.50) superfamilies. For example, in the P loop nucleotide hydrolase superfamily (3.40.50.300), all nonredundant relatives are structurally diverse (i.e., superposing with >5Å), and these domains occur in many different domain contexts (see Table S1). A total of 286 Gene Ontology (GO) functional terms can currently be identified for this superfamily, which gives some indication of its functional diversity. Figures 3A and 3B show an example of two diverse relatives from this superfamily, and Figure 3C illustrates that, although all relatives possess the same highly conserved structural core, there can be extensive structural embellishments between relatives. Additional examples from other diverse superfamilies are shown in Figures S7–S10, again highlighting the common core between relatives and different secondary structure decorations to this core.

**Figure 4:**
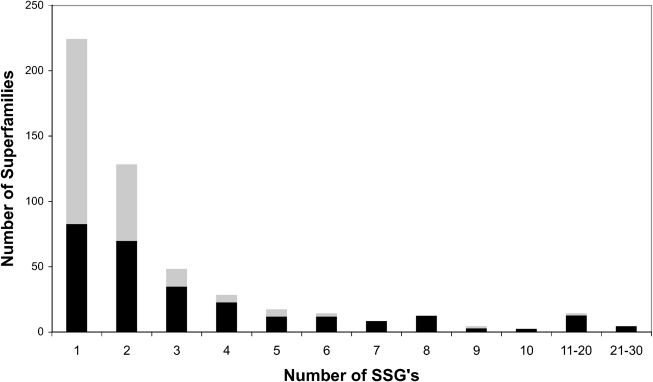
Relationship Between the Number of SSGs and Species Distribution The black regions represent the number of superfamilies that are universal to all species, whereas the gray regions represent all other superfamilies.

**Figure 5:**
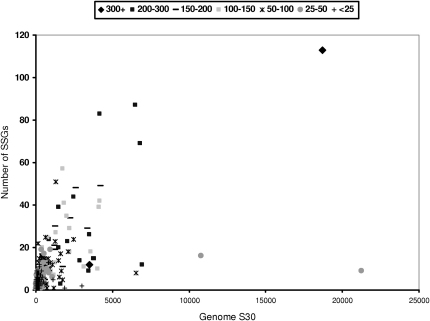
Correlation Between the Degree of Structural Diversity Across a Superfamily, Measured by the Number of SSGs and Population of the Superfamily, in Terms of Number of Sequences, in the Genomes (in Gene3D) The number of functions attributed to each superfamily is represented using symbols according to the number of FunCat categories.

Although there are only 4% of superfamilies in CATH with five or more SSGs, these superfamilies are very highly populated in the genomes, accounting for nearly 40% of predicted domain structures in the sequences of completed genomes in Gene3D, and most of them are universal to all kingdoms of life (Figure 4). Interestingly, if we use the threshold of <5Å to define SSGs, we observe 3118 SSGs in CATH. Twenty-five percent of all SSGs are identified in the 4% of highly diverse superfamilies. There is also clearly some correlation between the structural diversity exhibited by these superfamilies and their recurrence in the genomes and functional diversity (Figure 5).

### Structural Overlap Between Fold Groups

Another phenomenon challenging the CATH hierarchy is the existence of structural overlaps between different folds. Previously, we commented on a Russian doll effect whereby folds were linked by overlapping motifs (Orengo et al., 1997). Many similar arguments have appeared in the literature since then (Grishin, 2001; Krishna and Grishin, 2005; Kolodny et al., 2006; Sippl et al., 2008) supporting a more continuous relationship between structures in fold space for some types of structures. To examine quantitatively the extent to which this effect exists and determine whether it has become more pronounced following the expansion of some superfamilies with structurally diverse relatives, we applied the same criteria used to recognize structurally coherent groups in superfamilies, to recognize structures in different fold groups that were similar.

**Figure 6:**
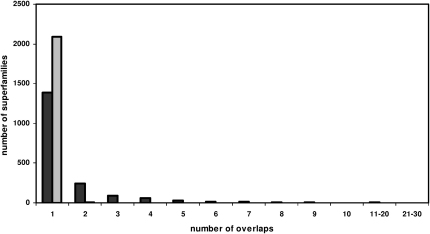
The Number of Superfamilies Displaying the Number of Overlaps with Other Superfamilies Each overlap corresponds to one or more domains in the particular superfamily overlapping with one or more domains in another superfamily. The black (gray) bar corresponds to overlaps where the residue overlap threshold is 60% (80%).

Specifically, to detect “structural overlap” between domains in different CATH fold groups, we identified cases where structures overlapped with a normalized RMSD <5Å. This criterion is the same one used to examine structural divergence in superfamilies. In addition, at least 60% of residues in the larger domain should overlap with residues in the smaller domain. This overlap constraint was imposed to ensure significant “fold” similarities between the domains as opposed to small “motif” similarities. For 76% of superfamilies, there was no overlap with structures in different fold groups (see Figure 6). This finding is perhaps not surprising given that most superfamilies currently exhibit little structural drift and hence are structurally coherent (see previous section).

For the remaining 24% of superfamilies that overlap with different fold groups, only 32% of the nonredundant relatives within these superfamilies are involved in the overlaps, and many of the overlaps disappear if more stringent thresholds are imposed (i.e., >80% residues in the larger domain can be superposed on the smaller domain with a normalized RMSD <5Å) (see Figure 6). Furthermore, most of the overlapping superfamilies comprise small domains containing fewer than six secondary structures (Figure S11a) or less than 80 residues (Figure S11b), and overlaps comprise super-secondary motifs (e.g., α-hairpins, αβ, and split αβ) that recur or comprise a large proportion of the fold.

Although 495 superfamilies are involved in overlaps, nearly half (48%) of all overlaps are associated with folds in the α-bundle and α-orthogonal architectures and involve superposition of an α-hairpin motif (see Figure S12). The highly recurrent αβ unit present in the Rossmann fold and other folds adopting αβ sandwiches is another common motif mediating structural overlaps (see Figure S13). Its recurrence is clearly one factor explaining the large number (61) of overlaps between the P loop nucleotide hydrolase superfamily and other 3 layer αβ superfamilies.

**Figure 7:**
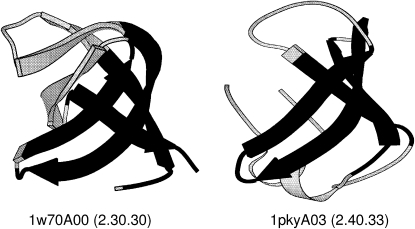
Structural Overlap (in Black) Involving Two Domains, One Possessing a β-Roll the Other a β-Barrel Architecture Normalized RMSD = 2.95. Residue overlap is 65%. Figure created using Molscript (Kraulis, 1991).

A large number of overlaps also feature small domains adopting β-roll architectures (2.30). In these very small domains, the overlap of a β-meander motif can constitute a very significant proportion of the domain structure (see Figure 7). The αβ-plait motif (Orengo and Thornton, 1993) is another small super-secondary structure overlapping frequently between different two-layer αβ folds. Domains containing these motifs are frequently small (<100 residues), and again a single αβ-plait motif can be a large part of the overall fold.

**Figure 8:**
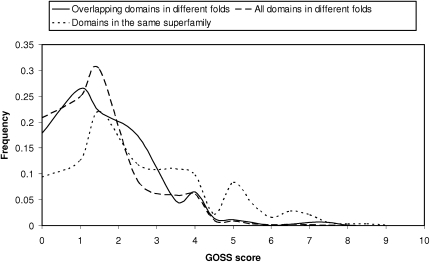
GOSS Scores for Overlapping Domains in Different Folds Compared to All Domains in the Same Superfamily and Also all Domains in Different Folds GOSS scores are obtained by comparing functional annotations from the gene ontology (GO) according to semantic similarity (see Experimental Procedures). A GOSS score of 5 and above is highly indicative of functional similarity.

Figure 8 shows that the existence of an overlap is rarely indicative of significant functional similarity between the domains. That is, the overlapping motifs are unlikely to be associated with recurrent functional motifs.

### How Does Superfamily Divergence and Fold Overlap Vary with the Normalized RMSD Threshold Used to Recognize Structural Similarity?

**Figure 9:**
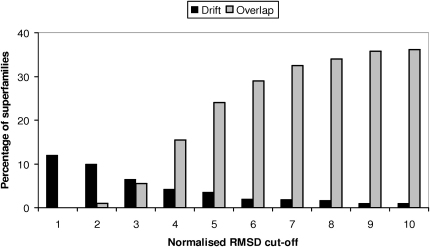
Plot Showing the Percentage of Superfamilies that Overlap (Gray) and Drift (>5 SSGs) (Black) for Different Normalized RMSD Cut-Offs

The extent of divergence within, or overlap between, superfamilies is clearly dependent on the thresholds used to recognize significant structural similarity. Figure 9 shows that as the threshold on the normalized RMSD is varied from 3 Å to 10 Å, the percentage of superfamilies significantly drifting (i.e., having five or more SSGs) and/or overlapping varies considerably. At a threshold of 3 Å, most superfamilies are observed to experience some structural drift but there is relatively little overlap. However, as the threshold is raised, the proportion of divergent superfamilies decreases (as the number of distinct SSGs with the superfamilies falls), while the structural universe as represented by CATH appears more as a continuum with significant numbers of superfamilies overlapping with other superfamilies. Similarly, as the threshold is varied between 3Å and 10Å, the number of SSGs identified varies from 7592 to 2380 (see Figure S14).

Using the threshold of a normalized RMSD of <5Å, the majority (32 of 40) of architectures exhibit no, or very few, overlaps. However, in eight architectures that are the most highly populated with sequences of completed genomes, there are structural overlaps between some fold groups.

**Figure 10:**
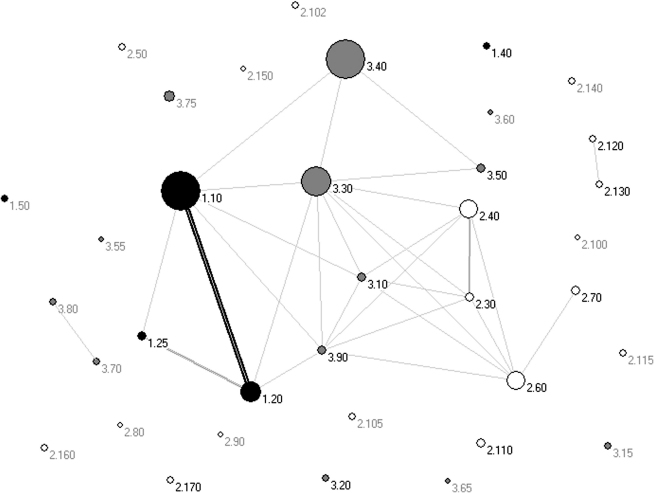
Network Plot Illustrating the Extent of Structural Overlap Between Different CATH Architectures Black, mainly α; white, mainly β; and gray. mixed α/β. Each point is labeled with its CATH architecture code in the form C.A. The thickness of the lines represents the number of overlapping superfamilies between the architectures. The size of the circles represents the number of sequence subfamilies (S35s, sequences clustered together at 35% sequence identity) in that architecture. Those architectures shown to overlap with at least one other in the CATH database are labeled as follows: 1.10 = α-orthogonal, 1.20 = α-up-down bundle, 1.25 = α-horseshoe, 2.30 = β-roll, 2.40 = β-barrel, 2.60 = β−sandwich, 2.70 = distorted β sandwich, 2.120 = β-6-propellor, 2.130 = β-7-propellor, 3.10 = αβ-roll, 3.30 = 2-layer αβ−sandwich, 3.40 = 3-layer(αβα) sandwich, 3.50 = 3-layer (ββα) sandwich, 3.70 = αβ-box, 3.80 = αβ-horseshoe, and 3.90 = αβ complex. Figure created using Pajek (http://vlado.fmf.uni-lj.si/pub/networks/pajek/sunbelt97/pajek.htm).

Overlaps can also be visualized as connected networks, with the thickness of the connection determined by the extent of overlap (see Figure 10). By depicting the structural universe in this way, we see that in each protein class there are many islands representing architectures containing highly distinct fold groups with no overlaps to other fold groups. However, there are also a few notably large clusters that have been attracted to each other by the overlap of common super-secondary motifs (e.g., the overlap of α-hairpins between structures in the 1.10/1.20 mainly-α architectures).

Therefore, whether it is sensible or useful to represent the current structural universe as captured by CATH as discrete islands, a structural continuum, or something in between depends on how the classification will be exploited or applied. These issues are considered in more depth below.

### To What Extent Does Superfamily Diversity and Fold Overlap Challenge a Hierarchical Classification of Domain Structures?

#### Handling Structural Drift in Superfamilies

The concept of a fold is clearly meaningful as it allows us to characterize the topological arrangements of secondary structures in a domain structure. Furthermore, fold similarity can be assessed quantitatively following superposition of domains. However, even if we applied a liberal threshold for recognizing similar folds (e.g., <5Å normalized RMSD), some large CATH superfamilies would effectively contain multiple fold groups. Since CATH traditionally places the T-level or fold group above the superfamily (H-level), this phenomenon could potentially break the CATH hierarchy or result in fragmentation of some superfamilies into multiple fold groups. However, if the superfamily is considered to be the major interest for biologists—this will certainly be the case for those exploiting the classification to understand protein evolution or infer function—homologs should be classified together in the same superfamily, despite structural variability.

It is possible to group homologs within the same H-level, and therefore T-level, if we consider structural similarity across the superfamily in the common domain core. As reported by Chothia and Lesk (1986) and still observed 20 years later with a much larger dataset (Reeves et al., 2006), there is considerable structural conservation in the evolutionary conserved domain core of homologs, which generally represents at least 40% of residues in the structure even in very divergent superfamilies. Furthermore, this topological core motif is likely to be structurally distinct from core motifs found in other superfamilies. In this sense, the hierarchical classifications of such resources as SCOP and CATH are still valuable if the fold group or topology level is thought of as grouping structures sharing similarities in their topological core motifs, where the core is the evolutionary conserved domain region of a superfamily.

The phenomenon of structural divergence has become more apparent over the last few years as a result of the development of highly sensitive sequence-based methods (profile-profile, HMM-HMM; see Reid et al., 2007 for review) that aid the detection of very remote homologs. It is this expansion of superfamilies with very diverse relatives that has highlighted the extreme structural plasticity of some domain superfamilies and the extent to which diverse structural decorations to the conserved core are tolerated.

Many of the highly divergent superfamilies adopt simple two and three layered (mainly-β and αβ) architectures. Previous analyses of 31 of these superfamilies (Reeves et al., 2006) demonstrated how the regular structural arrangements adopted in the conserved cores of domain relatives provide stable frameworks that can support a great variety of structural decorations. Most structures have central beta sheets, and since insertions are rarely tolerated in the core, they tend to occur in only a few positions on the domain surface—at the tops, bottoms, or edges of the beta sheet(s). This means that insertions accumulate at relatively few positions, giving rise to more dramatic structural changes.

Evolution is influenced by this tolerance to structural change. Paralogous relatives with structural variations that modify the active site or protein-protein interaction surfaces, thereby expanding the functional repertoire of the organism, are likely to be expressed and retained within the organism. The structural plasticity, therefore, provides some rationale for the wide expansion of these superfamilies in the genomes (Goldstein, 2008). Analysis of sequence diversity (Marsden et al., 2006) suggests that less than half the sequence diverse relatives in these superfamilies have been structurally characterized, which means that it is likely that additional SSGs will be identified in the future.

Since the divergent superfamilies account for nearly 40% of sequences in the genomes with predicted structures, it is important that structural classifications derive strategies for characterizing them. In CATH, this phenomenon will be managed by identifying the topology of the evolutionary conserved core motif shared by all relatives and the various secondary structure embellishments to this common core. To capture information on structural diversity, the number of diverse SSGs within each superfamily will be recorded, and Rasmol images for each SSG will be displayed, highlighting conserved secondary structures across the superfamily (i.e., the conserved core) and secondary structure embellishments to this core. An example of the additional information that will be presented in CATH to capture information on structural diversity across fold groups and superfamilies is given in http://beta.cathdb.info/cathnode/3.40.50.620 for a Rossmann fold superfamily. Details of the methods used to identify conserved and variable regions are presented on the same web site and also in our Supplemental Data.

#### Handling Overlap Between Fold Groups

When we consider the overlap between different superfamilies and fold groups, the data presented here suggest that, for some thresholds (e.g., 3 or 4 Å), there is little structural overlap between superfamilies and fold groups. When the RMSD threshold is relaxed to 5 Å or more, overlap is observed between superfamilies in some architectures (e.g., α-bundle, α-orthogonal, β sandwiches, and αβ sandwiches) often as a result of common super-secondary motifs. The frequent lack of any close functional relationship between the superfamilies that are overlapping suggests that these structural matches are more likely to be the result of physico-chemical constraints on folding or packing of the polypeptide chain—that is, convergence to a stable 3D arrangement. Although, as Lupas et al. (2001) and others have suggested, extremely distant evolutionary relationships based on these common motifs cannot be discounted.

Nearly half the overlaps involve common α-hairpins in superfamilies adopting α-bundle and α−orthogonal architectures. In addition, other small single super-secondary motifs overlap between domains (e.g, β-meanders). In this sense, fold space is perhaps better represented as a galaxy with dense and sparse clusters. However, some overlaps comprise larger motifs of four or more secondary structures, such as split αβ-motifs or recurring αβ-motifs. For these cases, it is possible to link from one fold to the next and to the next, via these motifs, as in a Russian doll effect, and this is more suggestive of a fold continuum. Superfamilies with no overlap at all tend to have very distinctive folds (e.g., the β-trefoil fold) comprising rather unusual motifs or unusual combinations of common motifs.

Although only 24% of superfamilies and less than 32% of the nonredundant structures within them (14% of all nonredundant structures in CATH) are involved in structural overlaps with different folds, these superfamilies account for a significant percentage of sequences in completed genomes. It is possible, therefore, that as more structures from these superfamilies are solved, more overlaps will be revealed. It is important that CATH should also reflect these lateral links, which traverse the traditional hierarchy. Consequently, in addition to the traditional hierarchical classification, CATH will also present horizontal links involving structural matches between different superfamilies and fold groups. Any significant structural overlaps between a domain and domains in different fold groups will be presented on the individual web page for that domain (see http://beta.cathdb.info/cathnode/3.40.50.620 for an example). In addition a matrix showing all overlaps between nonredundant representatives in CATH is downloadable from the CATH web site (http://release.cathdb.info/v3.1.0/structural_overlap_matrix.dat).

### Summary

A quantitative measure of domain structural similarity (<5 Å) has been used to explore structural diversity within CATH superfamilies and structural overlaps between fold groups. Using this measure, we observe that, in most superfamilies, domains tend to be structurally similar to other relatives. However, a small set of 78 superfamilies are highly divergent, comprising five or more distinct SSGs, where SSGs contain relatives superposing with a normalized RMSD of <5Å. Moreover, these superfamilies account for 25% of all SSGs identified in CATH superfamilies and are highly populated, accounting for nearly 40% of predicted domain structures in genome sequences.

A large proportion of superfamilies are structurally “distinct” from superfamilies in other fold groups. However, 24% show structural overlaps with other fold groups. While fewer than 32% of the nonredundant relatives within them are involved in overlaps, the superfamilies they belong to are highly populated with domain sequences in the genomes. Furthermore, since analysis of the genome sequences suggests that many more diverse relatives remain to be structurally characterized (Marsden et al., 2006), new structural data could subsequently identify additional overlaps. For these superfamilies, fold space should be viewed as more continuous.

## Experimental Procedures

### CATHsolid: The Hierarchical Organization of CATH

CATH is a hierarchical classification of protein domain structures according to sequence, structural, and functional similarity. Domains are initially sorted into four Classes by secondary structure content (mainly-α, mainly-β, mixed αβ, or few secondary structures). They are then classified according to their Architecture (arrangement of secondary structures in 3D, independent of their connectivity), then Topology/fold (where the connectivity between secondary structures are taken into account), followed by Homologous superfamily (where the domains share at least two out of the three following criteria; significantly similar in structure, significantly similar in sequence, and similar in function). Domains are also clustered into subfamilies with increasing sequence similarity (35%, 60%, 95%, or 100%, respectively). The term Sreps is used to describe domain representatives clustered at 35% sequence identity into S35 subfamilies.

### The CATH Update Protocol

There have been substantial developments in the CATH update protocol (Greene et al., 2007) (see Supplemental Data, section 8.1 and Figure S15) enabling a large increase in the numbers of structures classified over the last year. There are two major bottlenecks in the CATH update protocol—domain boundary assignment and domain homology classification. The aim has been to automate the assignment of domain boundaries and homologous relationships as much as possible, with manual curation only being necessary for the more challenging structures.

### Measuring Structural Drift Within CATH Superfamilies

Probably the best known method for measuring structural similarity is the root mean square deviation (RMSD) (Rossman and Argos, 1976). Structures are first aligned using the CATHEDRAL structure comparison algorithm (Redfern et al., 2007), and the alignment is used to guide a superposition of the domains using the McLachlan algorithm (McLachlan, 1982) in order to calculate RMSD. Since RMSD can be misleading if not used together with information on the number of aligned residues, we also use a normalized RMSD as proposed by Levitt and co-workers (Friedberg and Godzik, 2005; Subbiah et al., 1993). For generating coherent structural groups, it is valuable to consider an RMSD value normalized by the largest structure being compared. This is calculated as follows:

$$\text{Normalised}\;\text{RMSD}\;=\;\frac{\left(\text{maxlength}\right)\;\text{x}\;\text{RMSD}}{\text{N}},$$

where maxlength = number of residues in the largest structure, and N = total number of aligned residues. In this analysis, a normalized RMSD of less than 5 Å is taken as indicative of significant global structural similarity.

We examined the degree of structural divergence between close relatives in CATH S35 sequence families. It can be seen from Figure S16 that, for a significant majority of pairs within CATH S35 families, the structures are very similar with normalized RMSD below 5 Å. For this reason, and since accurate pair-wise structure comparison can be computationally very expensive, further investigations of structural drift in CATH superfamilies are conducted using a single representative domain from each s35 sequence family (Srep).

Figure S6 shows the distribution of pair-wise normalized RMSDs obtained from structural comparisons between Sreps in (a) the same superfamily, (b) the same fold group, and (c) different fold groups. It can be seen that the median value for the normalized RMSD between homologous relatives is ∼5 Å. Therefore, for the purpose of this analysis, the extent of structural drift within each superfamily was first assessed by considering the number of SSGs within a superfamily, where an SSG is generated by maximum linkage clustering of Srep relatives with a pair-wise normalized RMSD less than 5 Å to all other Sreps in the group. Structural drift was also calculated using a range of cutoffs on the normalized RMSD (4, 5, 6, 7, and 10 Å) to investigate the effect of varying this parameter on the resulting impression of fold space.

Figure S17 shows that the normalized RMSD between homologous domains are relatively independent of the average sizes of the domains. In order to remove any bias caused by highly populated superfamilies, representative pairs have been plotted for each CATH superfamily. The pairs with the smallest and largest normalized RMSD were selected.

### Measuring Structural Overlap Between CATH Superfamilies

A structural overlap score was calculated to assess whether protein domains from one superfamily were significantly structurally similar to domains in other fold groups or architectures. A CATHEDRAL structural comparison (Redfern et al., 2007) was performed between each Srep domain within a homologous superfamily and Sreps from all other CATH superfamilies. Normalized RMSD were then calculated following superposition of the domains using the McLachlan algorithm (McLachlan, 1982). Scores below a given threshold (e.g., 5 Å) and where at least 60% of the larger domain is aligned against the smaller domain, were taken as indication of a valid structural overlap between the different superfamilies. The proportion of residues aligned between the two domains is described as the overlap parameter. The threshold of 60% was chosen to ensure that superfamilies and fold groups overlapping shared a significant proportion of residues and represented fold overlaps rather than motif overlaps between domains.

A superfamily was considered to overlap another one if there was at least one overlap between Sreps observed. Structural overlaps were analyzed between different superfamilies within the same fold and between superfamilies in different folds and architectures.

Architectures 2.10, 2.20, 3.100, and 4.10 were omitted from both structural drift and overlap analyses as these are not well-defined architectures but collections of structures with irregular secondary structures arrangements. Superfamilies within these collections have small populations (<3 Sreps). Superfamilies from regular architectures that were not sufficiently well populated (i.e., containing less than 3 Srep representatives) were also omitted from the structural drift analysis. This gave 559 highly populated superfamilies (3 or more Sreps) used in this analysis.

### Foldspin Plots: Highlighting Common Secondary Structures and Structural Diversity across a Set of Structures

A new method (foldspin) for representing structural diversity across a superfamily was used to visualize diverse relatives from structurally divergent superfamilies. Foldspin selects the most representative Srep relative from the superfamilies (i.e., having the smallest cumulative normalized RMSD across all relatives) and then calculates the normalized RMSD between this relative and other relatives in the superfamily. A two-dimensional plot is then generated that presents diversity across the superfamily by radially drawing lines from the central representative so that the length is proportional to the structural distance from the superfamily representative. Selected relatives are visualized on the plot using the MOLSCRIPT program (Kraulis, 1991).

### Identifying Sequence Relatives for CATH Superfamilies in the Genomes and Calculating Functional Similarities

In 2002, a sister resource, Gene3D (Buchan et al., 2002), was established for CATH that captures information on domain sequences, from completed genomes, that are predicted to belong to CATH domain structure superfamilies. Further details on CATH domain predictions in Gene3D are described in section 8.1 of the Supplemental Data. Information from the FUNCAT (Ruepp et al., 2004) and GO databases (Ashburner et al., 2000) was used to examine functional variation within each superfamily. Sequences in FUNCAT and GO terms were aligned to PDB chains using a standard Needlemann and Wunsch algorithm, and annotation terms were transferred when a sequence identity of at least 80% was obtained over 80% of the PDB sequence length. Functional similarity was calculated between domains by comparing their GO terms using the Resnik scoring system (Resnik, 1999) as described by Lord et al. (2003).

## Supplemental Data

Document S1. Seventeen Figures, One Table, and Supplemental Methods
