# Supplementary material for: The CATH Hierarchy Revisited—Structural Divergence in Domain Superfamilies and the Continuity of Fold Space
Source: Structure. 2009 Aug 12;17(8-8):1051–62. doi: 10.1016/j.str.2009.06.015 (PMC2741583; doi:10.1016/j.str.2009.06.015)
Supplement: Document S1. Seventeen Figures, One Table, and Supplemental Methods [file mmc1.pdf]

Supplemental Data

The CATH Hierarchy Revisited—

Structural Divergence in Domain Superfamilies

and the Continuity of Fold Space

Alison Cuff, Oliver C. Redfern, Lesley Greene, Ian Sillitoe, Tony Lewis, Mark Dibley, Adam Reid, Frances Pearl, Tim Dallman,1 Annabel Todd, Richard Garratt, Janet Thornton, and Christine Orengo

Section 8.1. The CATH update protocol: Automatic Steps in the Classification of Domain Structures in the CATH Database

8.1.1. Selecting chains for classification

The SIFT program (Greene et al, 2007) is first run to select all chains suitable for classification in CATH. The structure must be resolved using a valid method (NMR A or X-Ray Crystallography with resolution no greater than 4Å). It should be at least 40 residues in length and the number of carbon atoms can not make up more than half of the structure.

8.1.2 Assigning domain boundaries

Those chains that are closely related (>80% sequence identity) to multidomain chains in CATH are automatically chopped using the in house algorithm Chopclose. This generates a structural comparison of the domains using CATHEDRAL (Redfern et al, 2007), to inherit domain boundaries and checks that at least 80% of the larger chain is aligned against the smaller chain and that no more than 10 residues are found on either end of the alignment.
If no close relative is found, manual curation is necessary and a consensus decision is made based on information given by several algorithms including CATHEDRAL (Pearl et al, 2003), SSAP (Orengo et al, 1996). DETECTIVE (Swindells, 1995), PUU (Holm et al, 1994). DOMAK (Siddiqui et al, 1995), Hidden Markov Models (HMM's) (Karplus et al, 1998) and the relevant literature. Information from these programs is collated on web pages (http://www.cathdb.info) which are now publicly accessible prior to full classification of the domain. CATHEDRAL (CATHs Existing Domain Recognition Algorithm) is a powerful new in-house automated domain boundary assignment prediction method which combines secondary structure matching to identify putative domain boundaries which are later refined using a slower, more accurate dynamic programming protocol (Redfern et al, 2007). A CATHEDRAL server has been set up and can be accessed from the CATH we site (http://www.cathdb.info/cgi-bin/CathedralServer.pl).

8.1.3 Classifying domains

In order for a particular structure to be classified within a given homologous superfamily at least two out of the three following criteria must be met

<u>Similar in Sequence</u>. Close relatives (structures with sequence identity of at least 35%) are identified using the Needleman and Wunsch algorithm (Needleman et al, 1970). More distant relatives are then identified using HMM based methods (Karplus et al, 1998) and PRC (http://supfam.org/PRC) to identify remote homologues. These methods are collectively referred to as HMMScan and their performance has been benchmarked on a manually curated consensus

CATH/SCOP dataset (Reid et al, 2007). Currently, at least 60% of remote homologues (<35% sequence identity) in CATH can be recognised for a reasonable error rate (1%) using the SAM-T single HMM method of Karplus et al (Karplus et al, 1998) and 80% using the PRC HMM-HMM method of Madera et al (Reid et al, 2007).

Similar in structure. CATH uses the SSAP (Orengo et al, 1996) and CATHEDRAL (Redfern et al, 2007) algorithms to perform structural alignments of protein domains. Both methods apply a logarithmic scoring scheme normalized to be between 0 and 100, independent of protein size. Benchmarking has shown that comparing two proteins with similar folds generally results in scores of 70 or more and homologous proteins will often give scores of 80 or more. Once the two proteins have been structurally aligned they are superimposed via the McLachlan algorithm (McLachlan, 1982) and a normalized RMSD is calculated (Redfern et al, 2007).

Similar in function. Orthologous proteins are likely to have very similar functions. Paralogous arising from gene duplication may have diverged in function, however, analyses suggest that some aspects of molecular function may remain conserved e.g. sites on the surface used in catalysis or binding or intermediates formed during a catalytic reaction (Todd et al, 2001; Rison et al, 2002). Information is extracted from the public databases GO (Ashburner et al, 2000), COGs (Tatusov et al, 2003), EC (Bairoch, 2000), FunCat (Ruepp et al, 2004) and KEGG (Kanehisa et al, 2000) and also from the literature. The SAWTED algorithm (MacCallum et al, 2000) is used to compare SwissProt keywords between proteins and a method for comparing GO terms is also employed (Bairoch, 2000; Lord et al, 2003).

The SAM-T technology of Karplus et al (Karplus et al, 2005) is used to build superfamily specific Hidden Markov models (HMMs) from diverse representatives within the superfamily (Sreps). Sequences from completed genomes are scanned against the CATH Hmm library to recognise domains which can be assigned to CATH structural superfamilies. Release 3.1 has HMM models for 9000 S35 families. The latest release of Gene3D (release 6) contains 527 completed genomes from all kingdoms of life. Table S1 gives the population of each CATH superfamily following the assignment of Gene3D sequences. Gene3D also contains information on the functions of sequences containing predicted CATH domains.

Automatic homology assignment is only applied for close homologues (>= 80% sequence identity >= 60% of residues in larger domain aligned against the smaller domain.). For more remote homologues the automatic methods are applied as described above and the data generated by these methods together with information obtained from the literature is taken into account in a manual validation of the classification by he CATH curators.

Section 8.2. Methods for Identifying Consensus ‘ Core Motifs’ for Domain Superfamilies and Capturing the Structural Diversity in CATH

8.2.1 What is an SGG?

A Structural Sub-Group (SSG) describes a cluster of two or more CATH domains that have been grouped together due to their close structural similarity. The measure used to describe structural similarity is the SIMAX score which is the RMSD of the two aligned structures normalized by the ratio of the number of residues in the larger domain to the number of aligned residues (see Section 5.3). All comparisons within a SSG cluster have a SIMAX score of less than a

given cutoff. The cluster type SSG5 corresponds to domains that all have a SIMAX similarity score of <5Å.

In order to remove redundancy, SSGs are created from sequence family (S35) representatives. A cutoff of 35% sequence identity is used for selecting representatives since above 35% identity relatives are likely to share similar structures. Superfamilies with only one sequence family will not have any SSG clusters nor will SSGs be created from clusters with only one structure (called singletons).

8.2.2. What is a aggregate alignment/SSGA?

The SSGs provide tightly clustered groups of protein structural domains where all members of a given group are guaranteed to have close structural similarity. However, in order to investigate more distant evolutionary relationships, it may be useful to examine structural alignments between proteins with more diverse structural relationships. The structural sub-group aggregates (SSGA) provide a means of linking the tightly clustered SSGs together to examine more distant evolutionary relationships.

SSGAs are created in two steps. The first step selects representative structures from each SSG where the most representative structure for a given SSG is defined as the domain with the highest structural similarity to domains in neighbouring SSGs. The second step clusters these representative structures with a more relaxed structural similarity cutoff to allow a certain amount of structural diversity, but not so much as to make the structural alignment meaningless.

The SSGA9 aggregate cluster type selects one representative structure from each SSG5 cluster, then clusters these structures into one or more groups with a SIMAX cutoff of <9Å.

8.2.3. Identifying the core residues common to all relatives in an SSG or SSGA

The CORA multiple alignment algorithm was used to create structure-based alignments for the domains in both the SSG and SSGA clusters. A consensus score was then calculated for each alignment position as a simple ratio of the number of structures aligned at this position divided by the total number of structures in the alignment. Alignment positions that have a consensus score of 1.0 (i.e. positions where all structures could be aligned) were considered to contain “core” residues.

Highlighting the corresponding “core” residues for each structure in the SSG provides a means of examining the structural embellishments observed within SSG clusters (especially within the more disparate SSGS clusters). It also allows comparison of common cores between superfamilies and the manual clustering of superfamilies sharing similar common cores into the same (T)-level (i.e., (T)opological core motif level) in CATH. Links have been provided on the web pages that allow the user to view the core residues highlighted on each domain in the SSGs through interactive molecular viewers (e.g., Jmol and Rasmol).

SUPPLEMENTAL REFERENCES

Bairoch, A. (2000). The ENZYME database in 2000. Nucleic Acids Res. *28*, 304–305.

Holm, L., and Sander, C. (1994). Parser for protein folding units. Proteins *19*, 256–268.

Kanehisa, M., and Goto, S. (2000). KEGG: Kyoto Encyclopedia of Genes and Genomes. Nucleic Acids Res. *28*, 27–30.

Karplus, K., Barrett, C., and Hughey, R. (1998). Hidden Markov models for detecting remote protein homologies. Bioinformatics *14*, 846–856.

Karplus, K., Katzman, S., Shackleford, G., Koeva, M., Draper, J., Barnes, B., Soriano, M., and Hughey, R. (2005). SAM-T04: what is new in protein-structure prediction for CASP6. Proteins. *61*, 135–142.

MacCallum, R.M., Kelley, L.A., and Sternberg, M.J.E. (2000). SAWTED: Structure Assignment With Text Description—Enhanced detection, of remote homologues with automated SWISS-PROT annotation comparisons. Bioinformatics *16*, 125–129.

Needleman, S.B., and Wunsch, C.D. (1970). A general method applicable to the search for similarities in the amino acid sequence of two proteins. J. Mol. Biol. *48*, 443–453.

Orengo, C.A., and Taylor, W.R. (1996). SSAP: sequential structure alignment program for protein structure comparison. Methods Enzymol. *266*, 617–635.

Pearl, F.M., Bennett, C.F., Bray, J.E., Harrison, A.P., Martin, N., Shepherd, A., Sillitoe, I., Thornton, J., and Orengo, C.A. (2003). The CATH database: an extended protein family resource for structural and functional genomics. Nucleic Acids Res. *31*, 452–455.

Rison, S.C., Teichmann, S.A., and Thornton, J.M. (2002). Homology, pathway distance and chromosomal localization of the small molecule metabolism enzymes in Escherichia coli. J. Mol. Biol. *318*, 911–932.

Siddiqui, A.S., and Barton, G.J. (1995). Continuous and discontinuous domains: an algorithm for the automatic generation of reliable protein domain definitions. Protein Sci. *4*, 872–884.

Swindells, M.B. (1995). A procedure for the automatic determination of hydrophobic cores in protein structures. Protein Sci. *4*, 93–102.

Tatusov, R.L., Fedorova, N.D., Jackson, J.D., Jacobs, A.R., Kiryutin, B., Koonin, E.V., Krylov, D.M., Mazumder, R., Mekhedov, S.L., Nikolskaya, A.N., et al. (2003). The COG database: an updated version includes eukaryotes. BMC Bioinformatics *4*, 41.

Todd, A.E., Orengo, C.A., and Thornton, J.M. (2001). Evolution of function in protein superfamilies, from a structural perspective. J. Mol. Biol. *307*, 1113–1143.

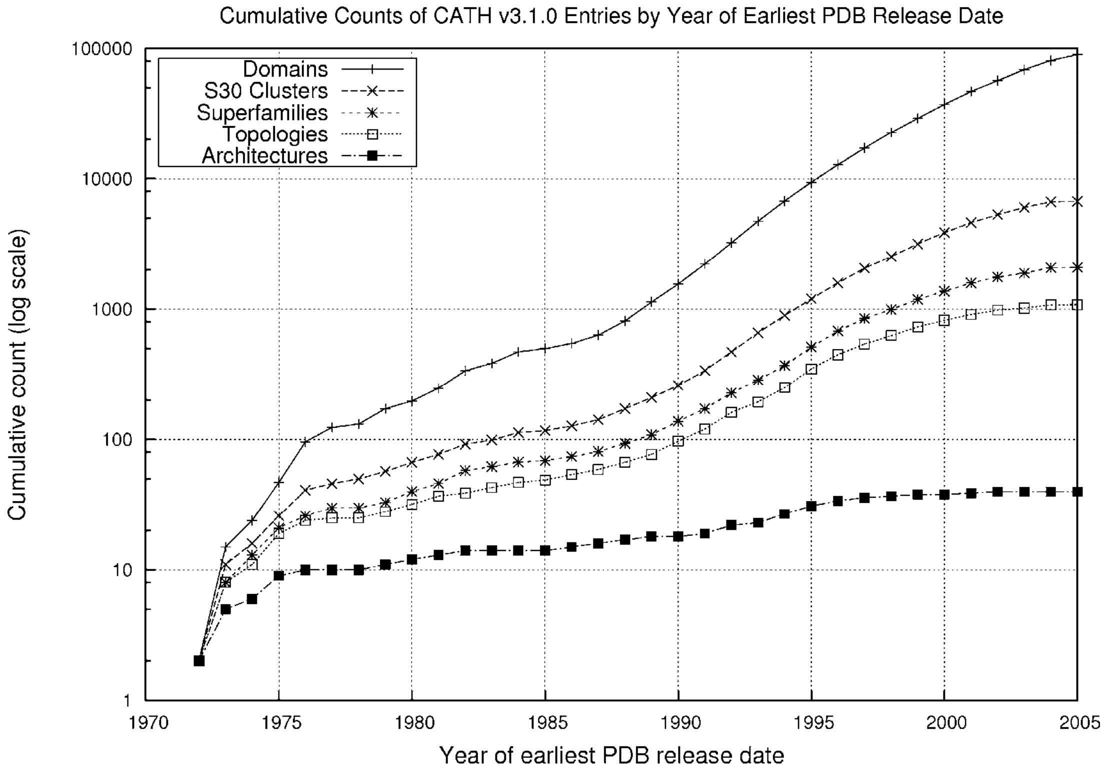

Figure S1.
Log Plot showing the growth of CATH from the beginning of the PDB to 2007 illustrating the increase in the number of domains, architectures, topologies, superfamilies and s30 clusters in the database.

A

Architectures in CATH version 3.1 (mostly α helical)

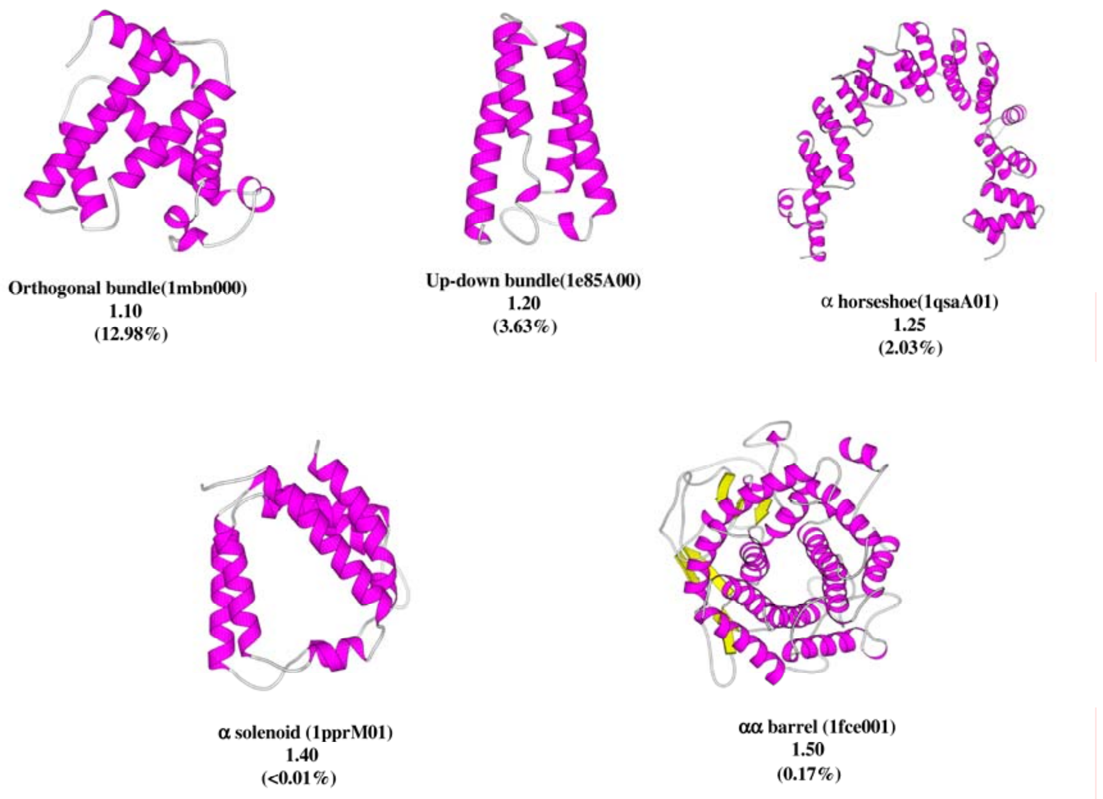

Architectures in CATH version 3.1 (mostly β sheet)

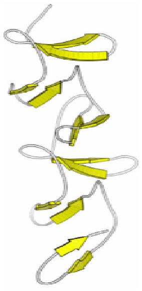

Ribbon (2bmlA00)
(0.88%)

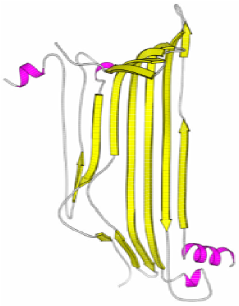

Sheet (1lshA03)
(0.34)

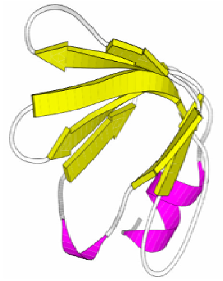

β Roll (1h64A00)
(1.94%)

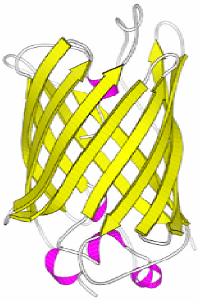

β barrel
(2fgqX00)
(3.68%)

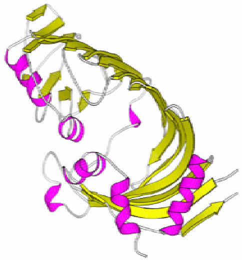

Clam (4bcl000)
(0.04%)

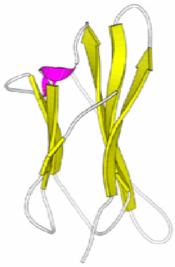

2-layer β Sandwich (1k5nA02)
(4.90%)

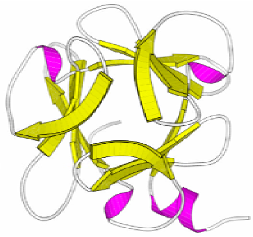

Trefoil
(1ybiA01)
(0.07%)

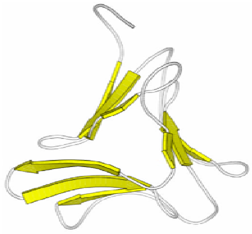

Orthogonal β-prism (1b2pA00)
(0.01%)

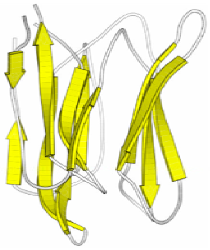

Parallel β-prism
(1ouwA00)
(0.01%)

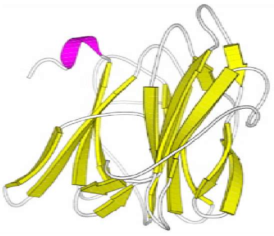

3-layer β sandwich
(1tg7A02)
(0.13%)

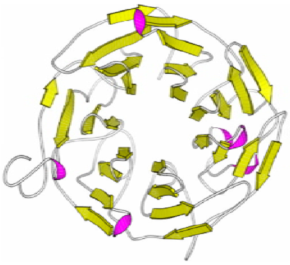

β propeller
(1k3iA02)
(1.29%)

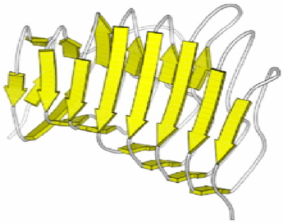

Solenoid (1ee6A00)
(0.44%)

Architectures in CATH version 3.1 (mixed α/β in structure)

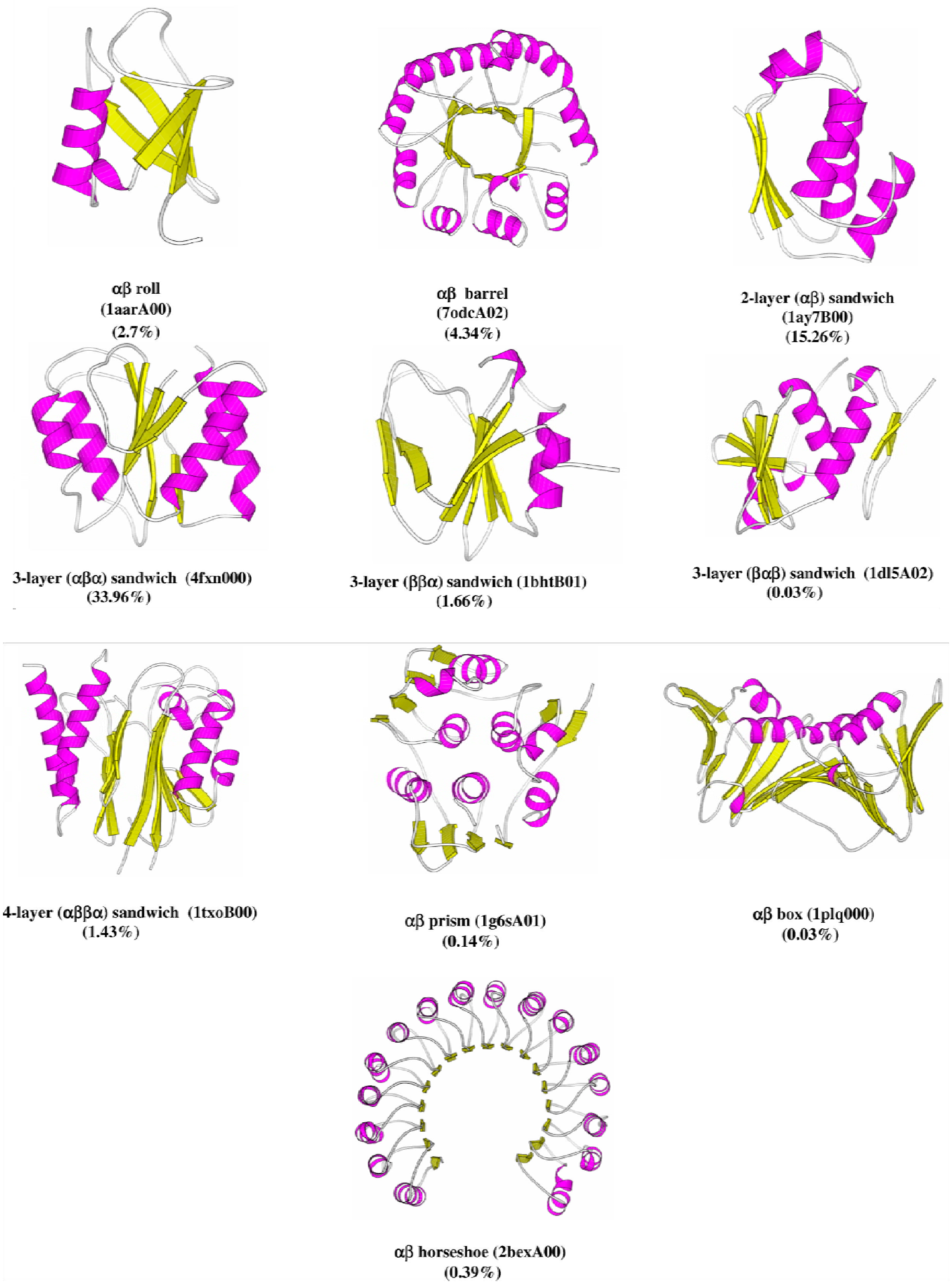

Figure S2.
Representatives from each architecture in CATH (v3.1). a) Mainly-α; b) Mainly-β; c) Mixed α/β. The number in brackets gives the percentages of non-redundant sequences (at 30% sequence identity) in Gene3D for each architecture.

The columns of the table are based on domain architectures as defined by the CATH hierarchical classification. Each cell provides information on an interesting fold group within that architecture and highlights a particular structural domain from that group. The first row of each column typically contains the most basic fold group for that architecture followed by fold groups with more complex structures. The population given as a percentage for each architecture is calculated from the 527 genomes present in Gene3D version 6.0.

Known functions have been automatically assigned to one of eight categories in the Gene Ontology (GO) molecular function classification (see legend). These categories are represented as a coloured octagon around the structure and are based on a classification scheme devised by Christos Ouzounis. For each fold group, the GO categories are those identified for all structures within that fold group, excluding electronically inferred annotations, as well as all annotated sequence homologues to those structures (at 60% sequence identity, 80% overlap of the larger domain) in Gene3D. Functions are assigned based on the whole structure to which the domain belongs and may therefore not always represent a specific functional attribute of that domain.

A white octagon tile means that no proteins in that fold group have that function. The incremental filling of the tile (by 1/4, 1/2, 3/4, 1/1) indicates the presence of the respective functions in the fold group and their relative importance (i.e. up to 25 / 50 / 75 / 100% of all proteins in that fold group have that function). For the fibrous proteins the functional mapping is simply that of the particular structure shown and its 60% homologues. A completely blank octagon reflects the fact that currently no function can be automatically mapped to that fold, but not necessarily that no known function exists.

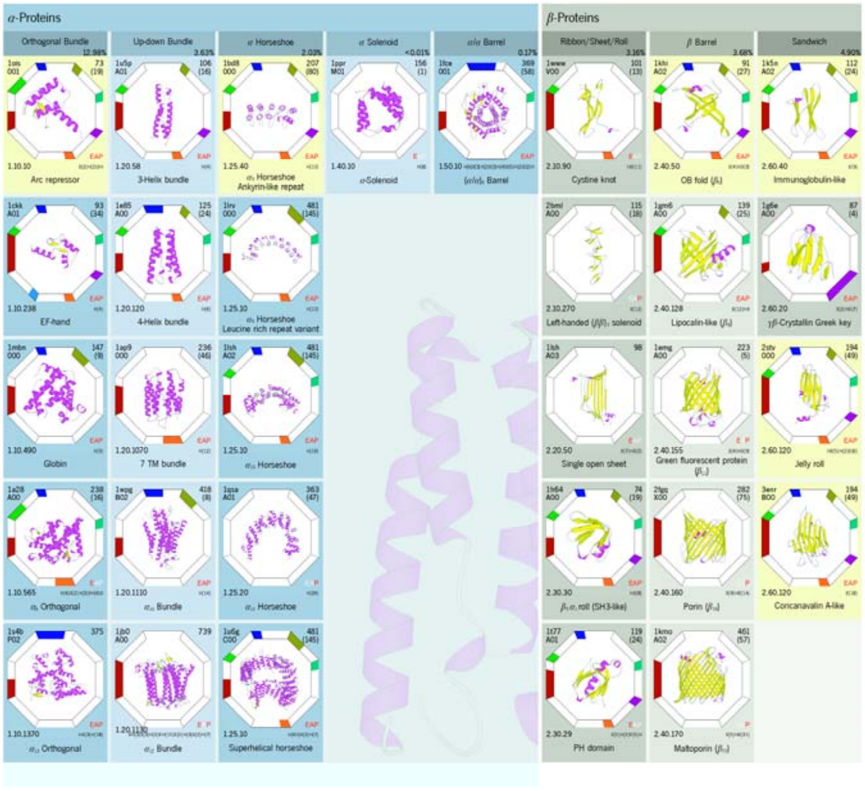

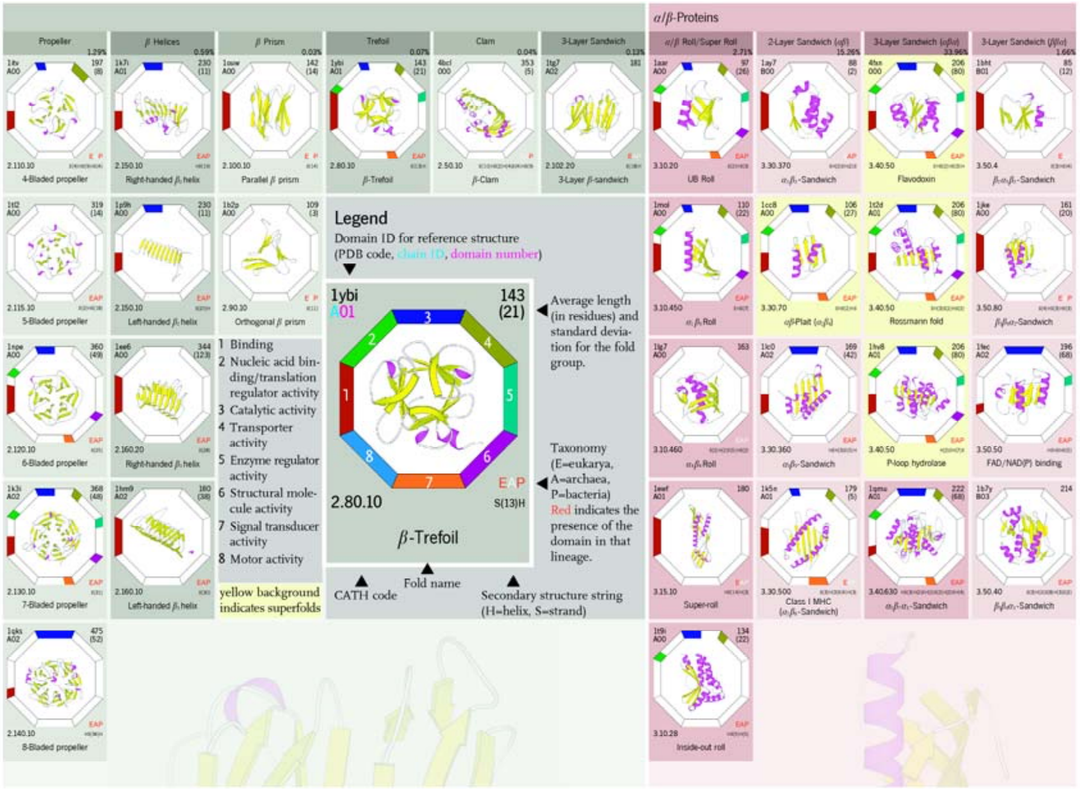

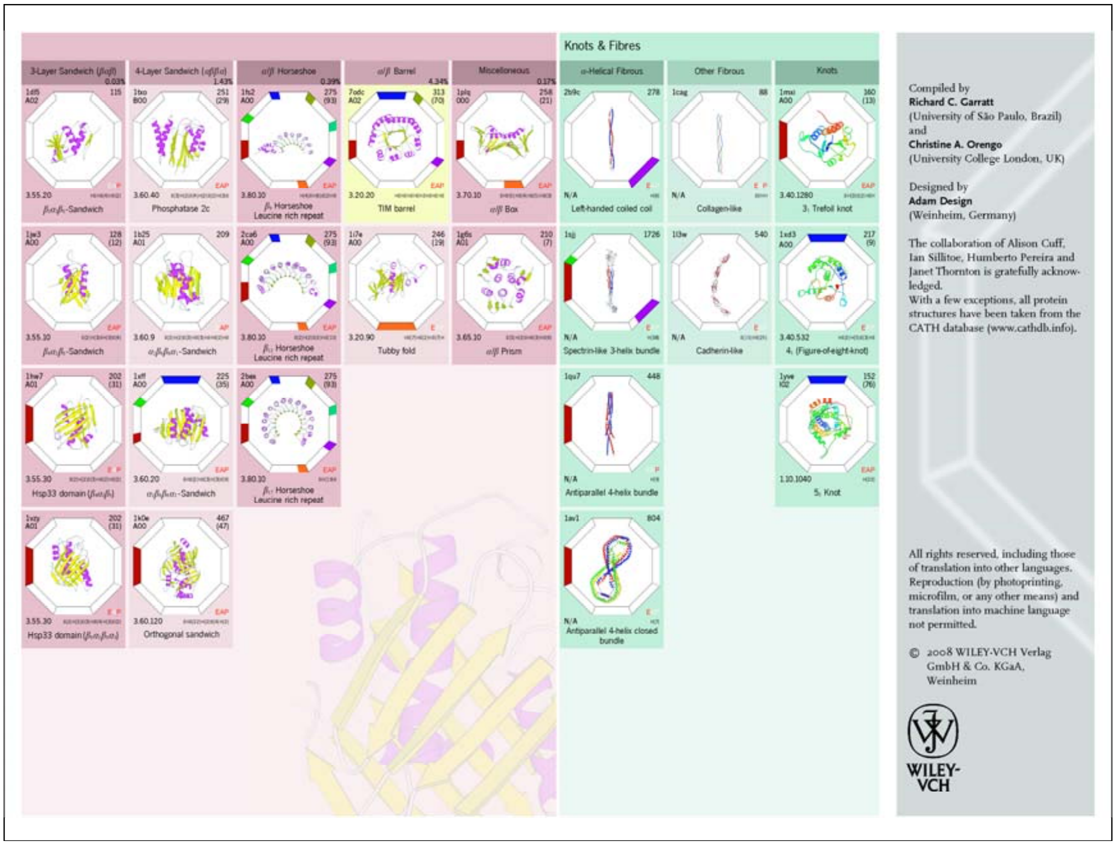

Figure S3. Snapshots of the Protein Chart (www.wiley.com)
A shows fold groups from the α architectures and some β architectures (B) fold groups from more β architectures and some αβ architectures and (C) more fold groups from αβ architectures (as well as some interesting knots and fibres).

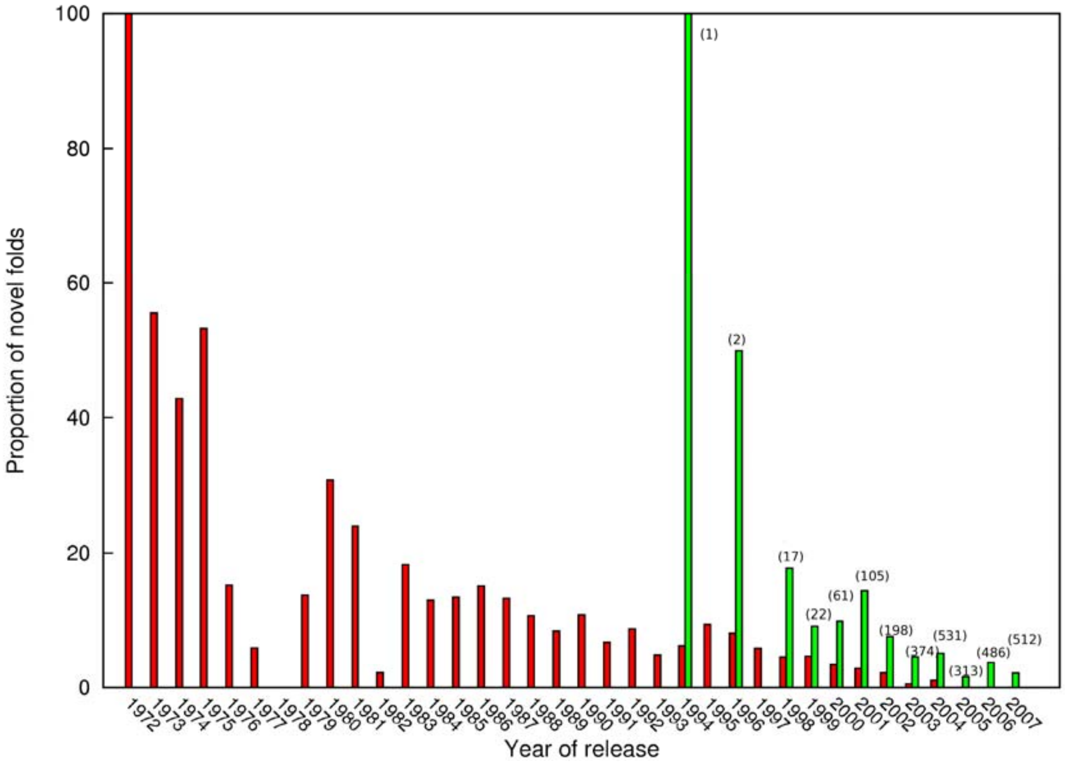

Figure S4. Proportion of new structures adopting novel folds (shown as a percentage for that year), as classified in CATH
The year refers to PDB release date. Red (green) bars indicate the proportion of novel folds determined by conventional structural biology (structural genomics initiatives). The numbers correspond to the number of SG structures deposited in the PDB for that year. SG structures were identified by their annotations in the PDB.

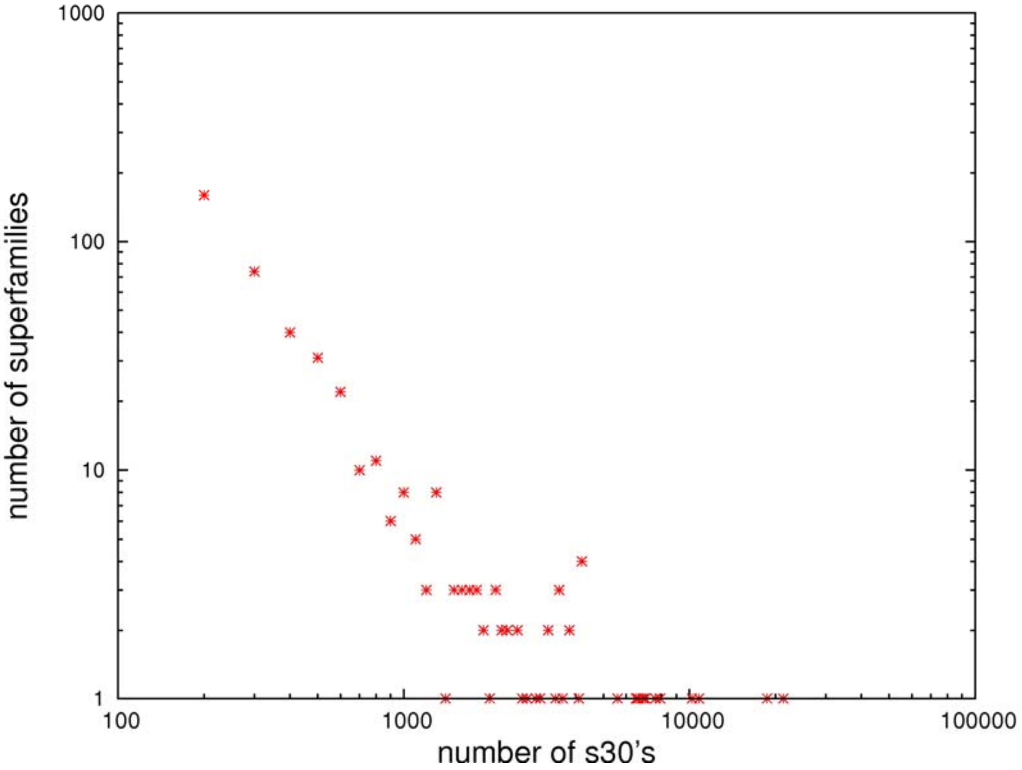

Figure S5.
Power law plot showing the frequency of superfamilies versus their population in the genomes (as number of s30s - relatives clustered at 30% sequence identity).

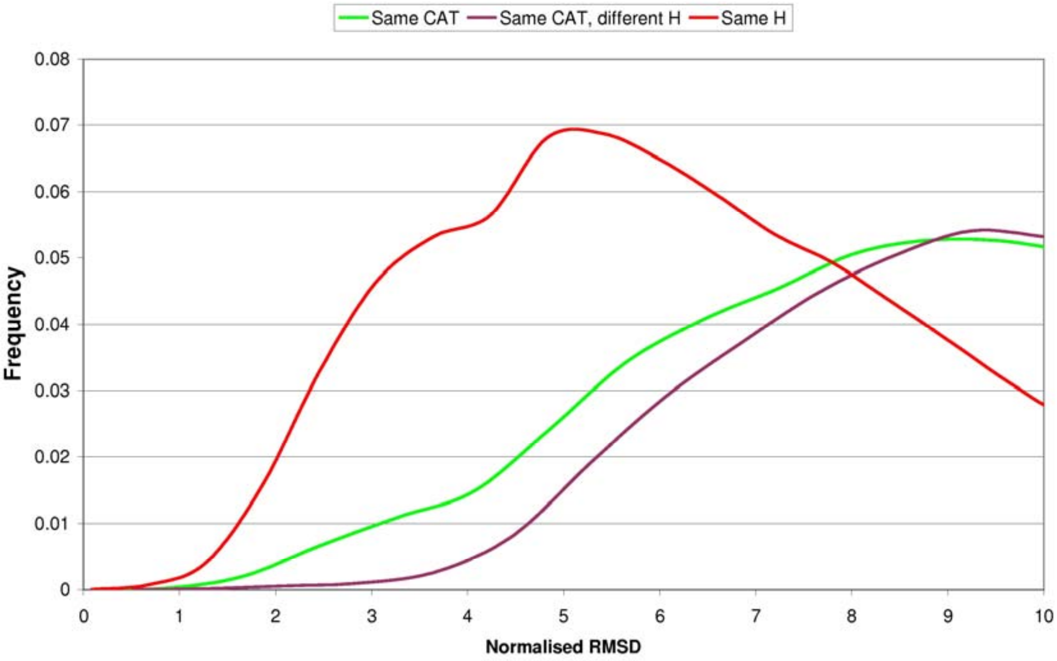

Figure S6.
Plot showing the distributions of pair-wise normalized RMSD scores obtained from structural comparisons between the same superfamily, same fold group, and between different fold groups.

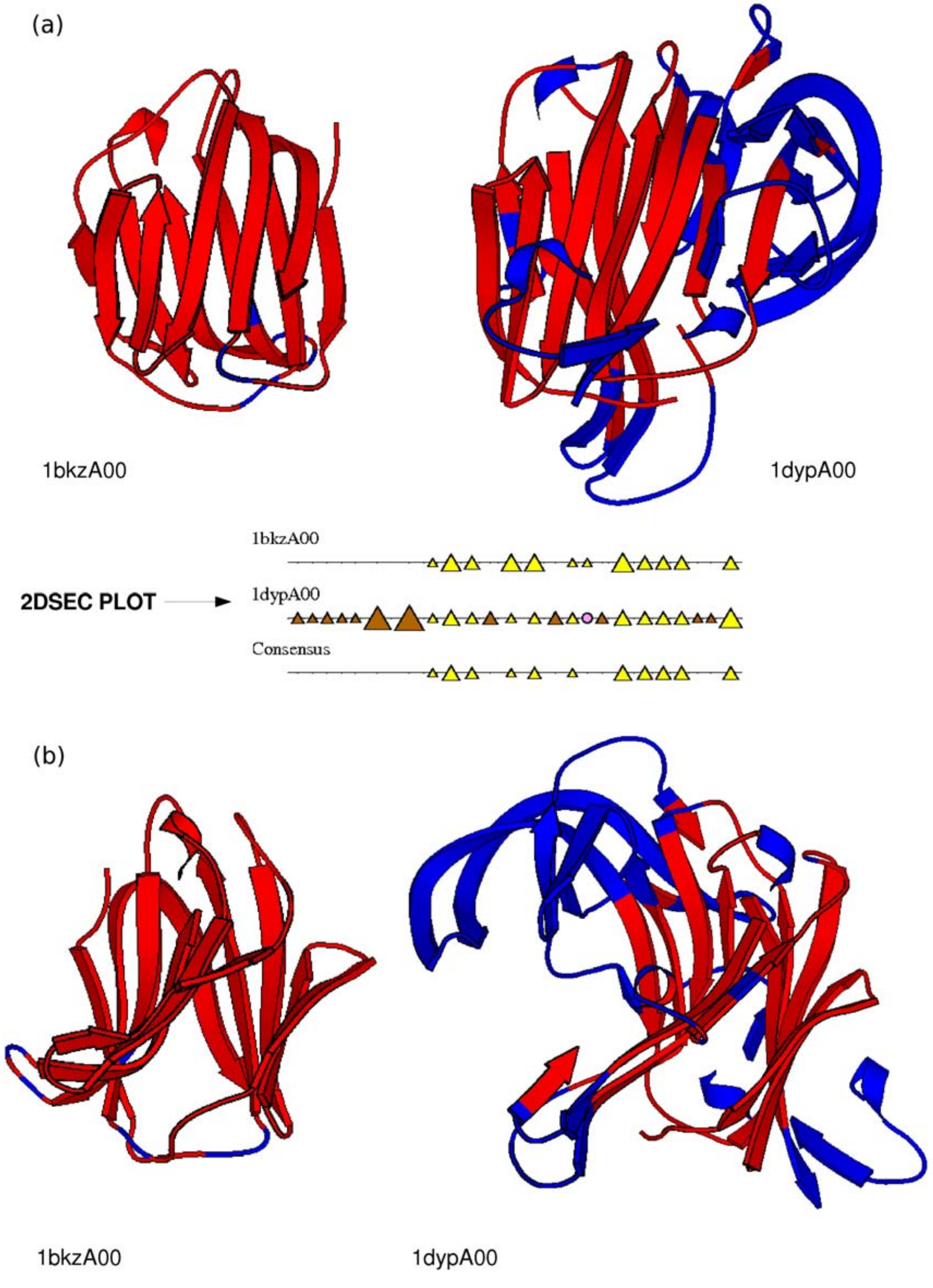

Figure S7.
(A) Common core (in red) and structural diversity (in blue) between two protein domains in the Galectin binding superfamily (2.60.120.200). The corresponding 2DSEC plot shows the secondary structure elements in the two domains and the features which are common to both structures (normalized RMSD = 7.25Å).
(B) side on view of the structural diversity between the structures shown in A.

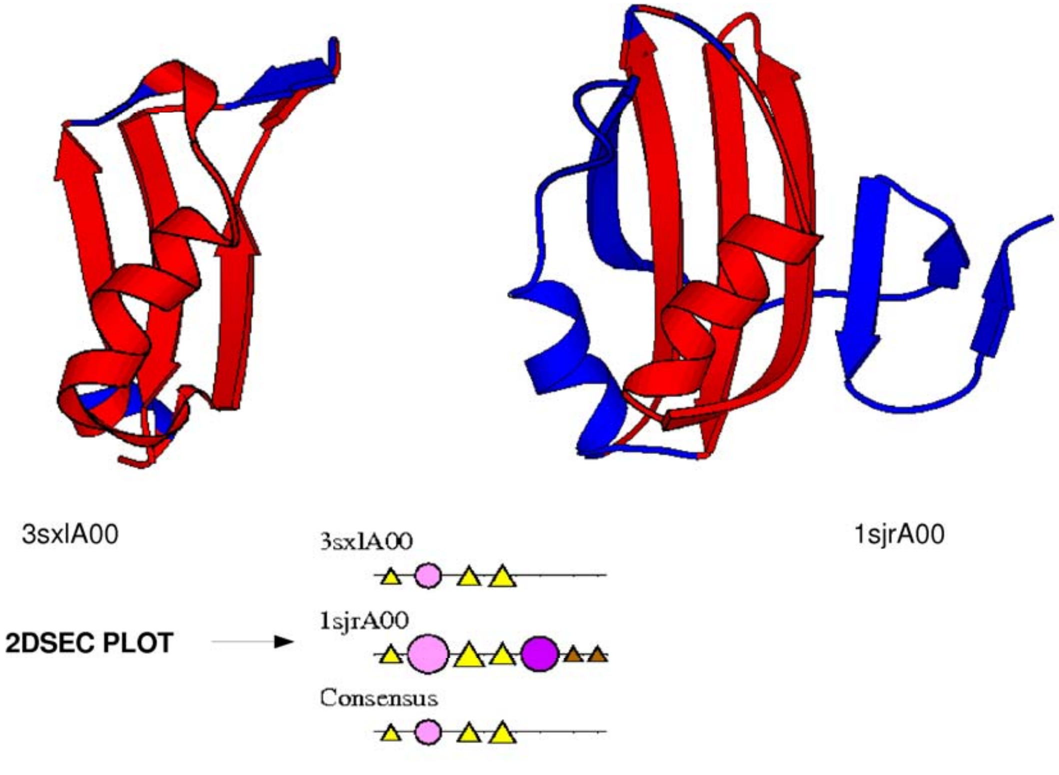

Figure S8.
Shows the structural variation between two α-β plait protein domains in the superfamily 3.30.70.300 (normalized RMSD = 5.60 Å).

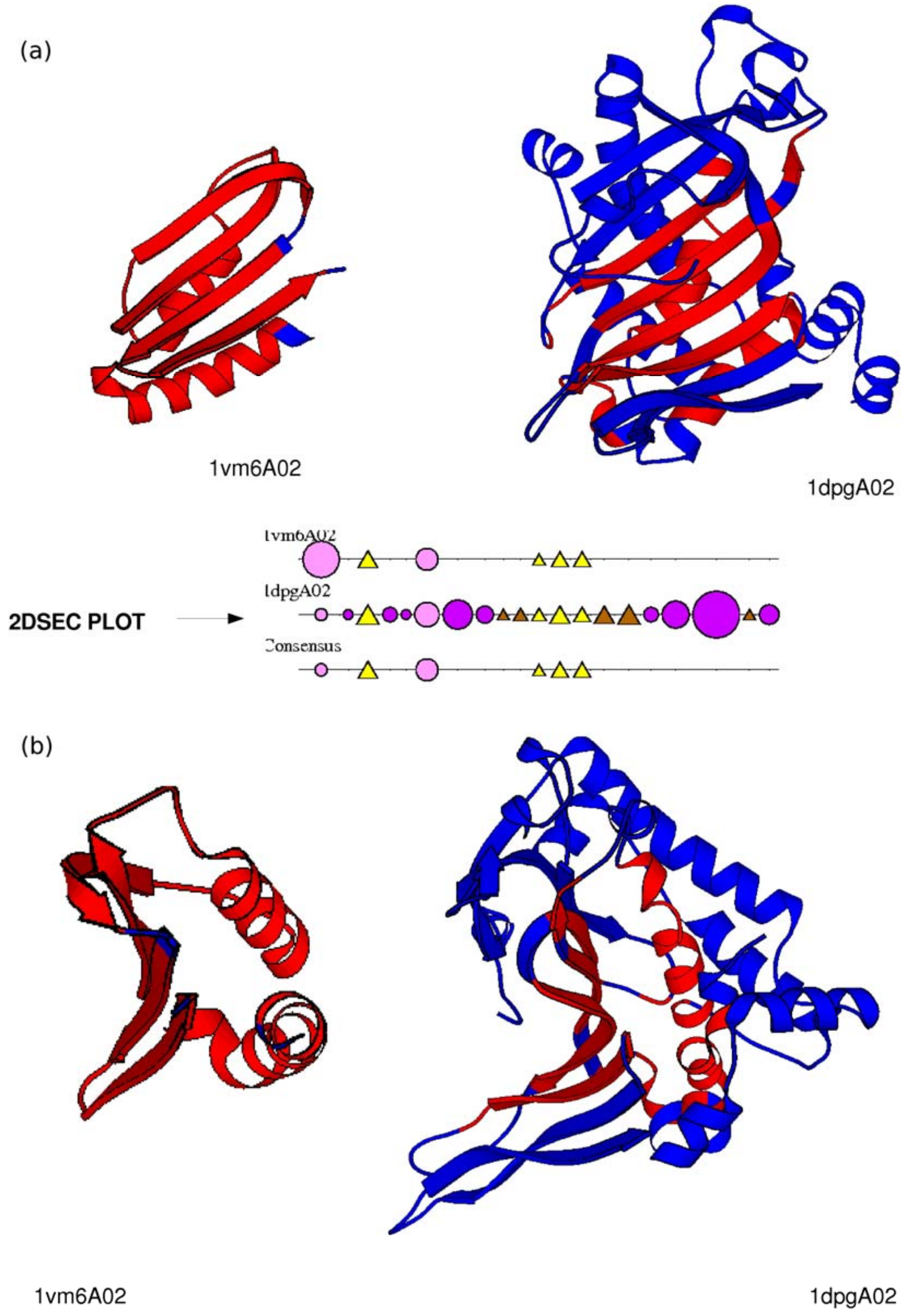

Figure S9.
A shows the structural variation between two protein domains in the NADP oxidoreductase superfamily by means of both molscript representations and a 2DSEC plot. Normalized RMSD = 15.68 Å. B gives a side on view of the domains in A.

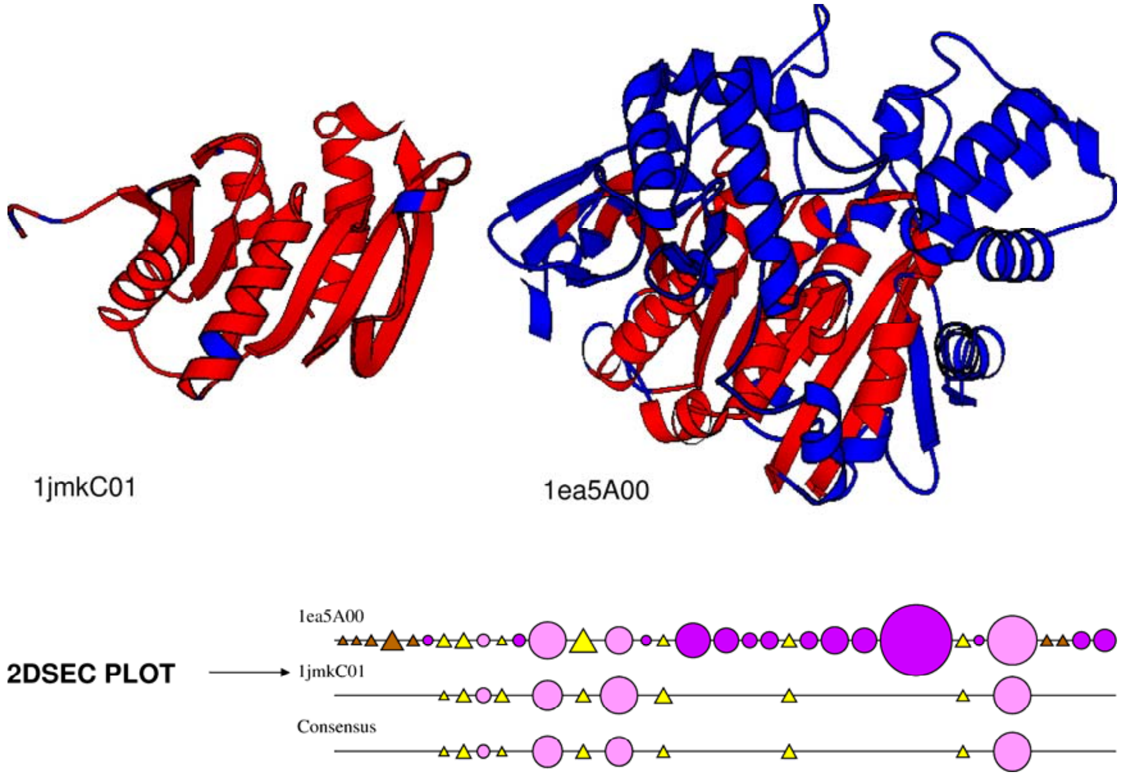

Figure S10.
Shows the structural variation between two domains in the αβ-hydrolase superfamily by means of both molscript representations and a 2DSEC plot. Normalized RMSD = 12.26 Å.

a)

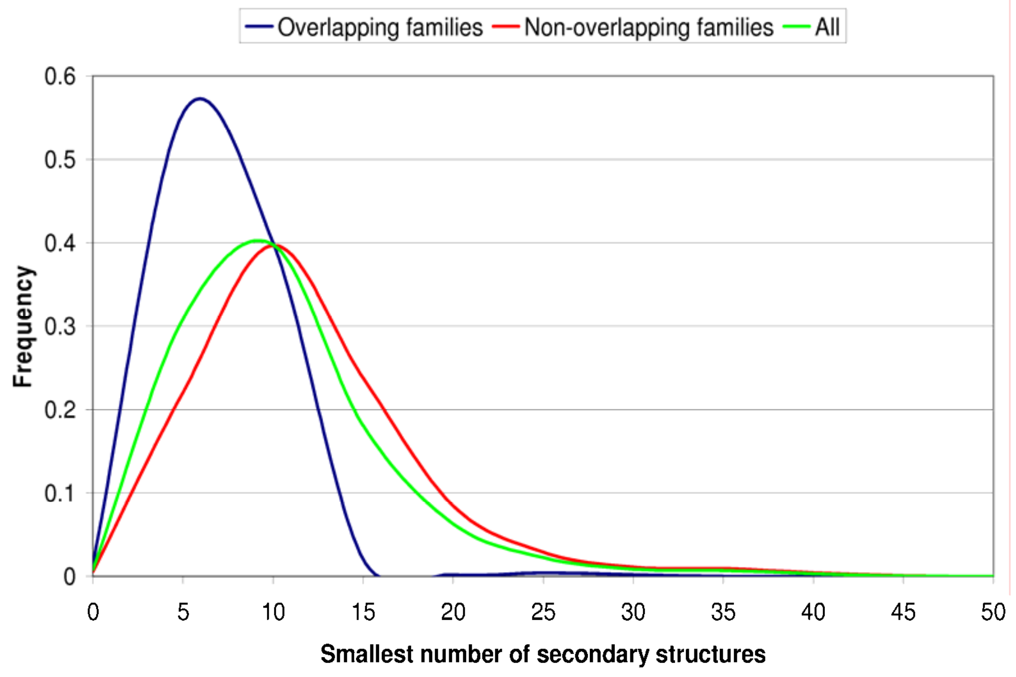

b)

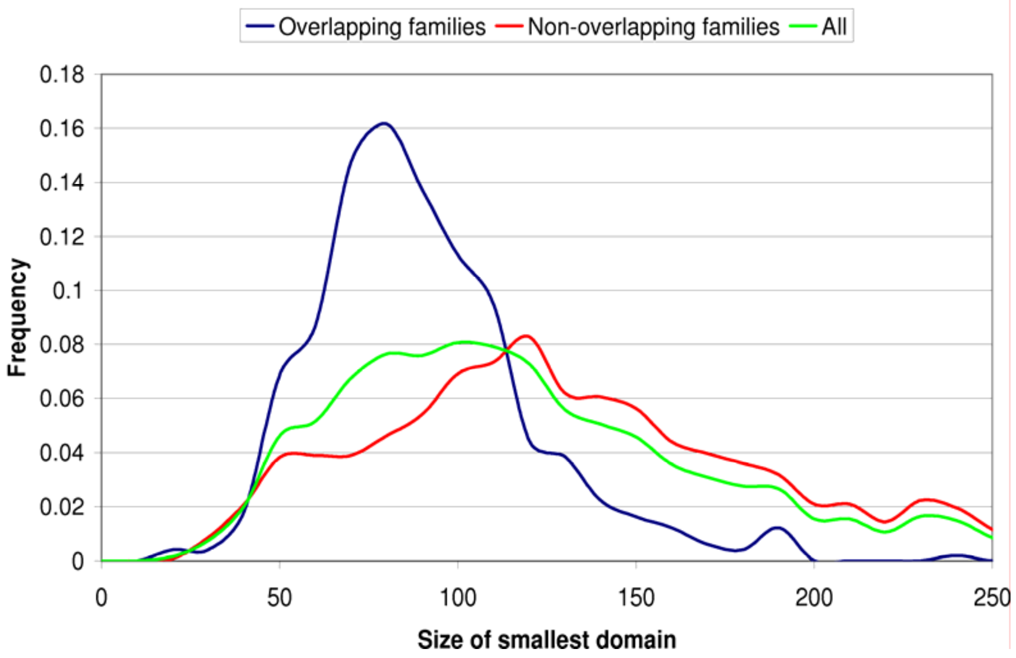

Figure S11.
(A) Plot showing the distribution of the size of the smallest domain (by number of secondary structures) for those superfamilies that overlap with other superfamilies and those that do not. It can be seen that overlapping superfamilies tend to comprise smaller domains.
(B) The same distribution as A, except the size of the smallest domain was calculated by the number of residues.

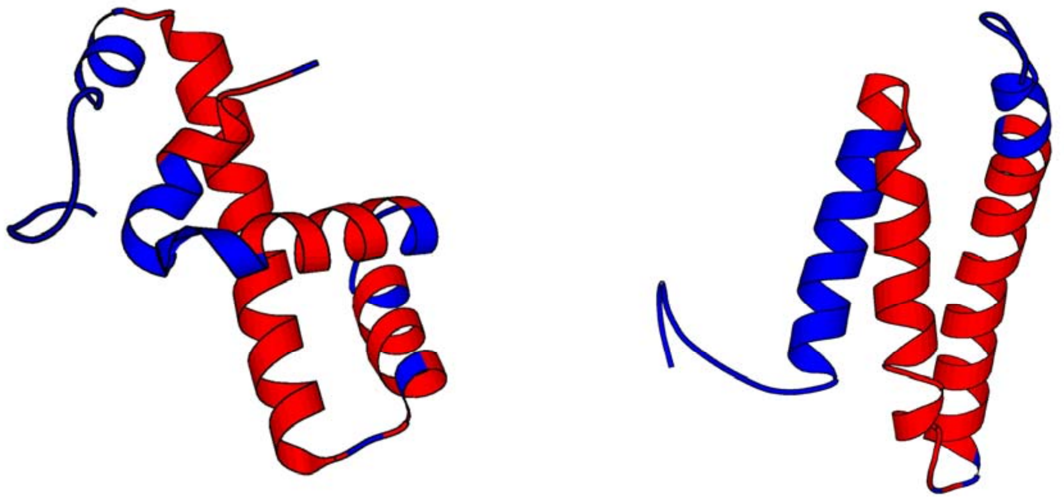

2ezkA00 (1.10) 1wfdA00 (1.20)

Figure S12.
Figure showing structural overlap between domains from an α bundle and an α orthogonal architecture. Normalized RMSD = 4.21Å and residue overlap is 81%.

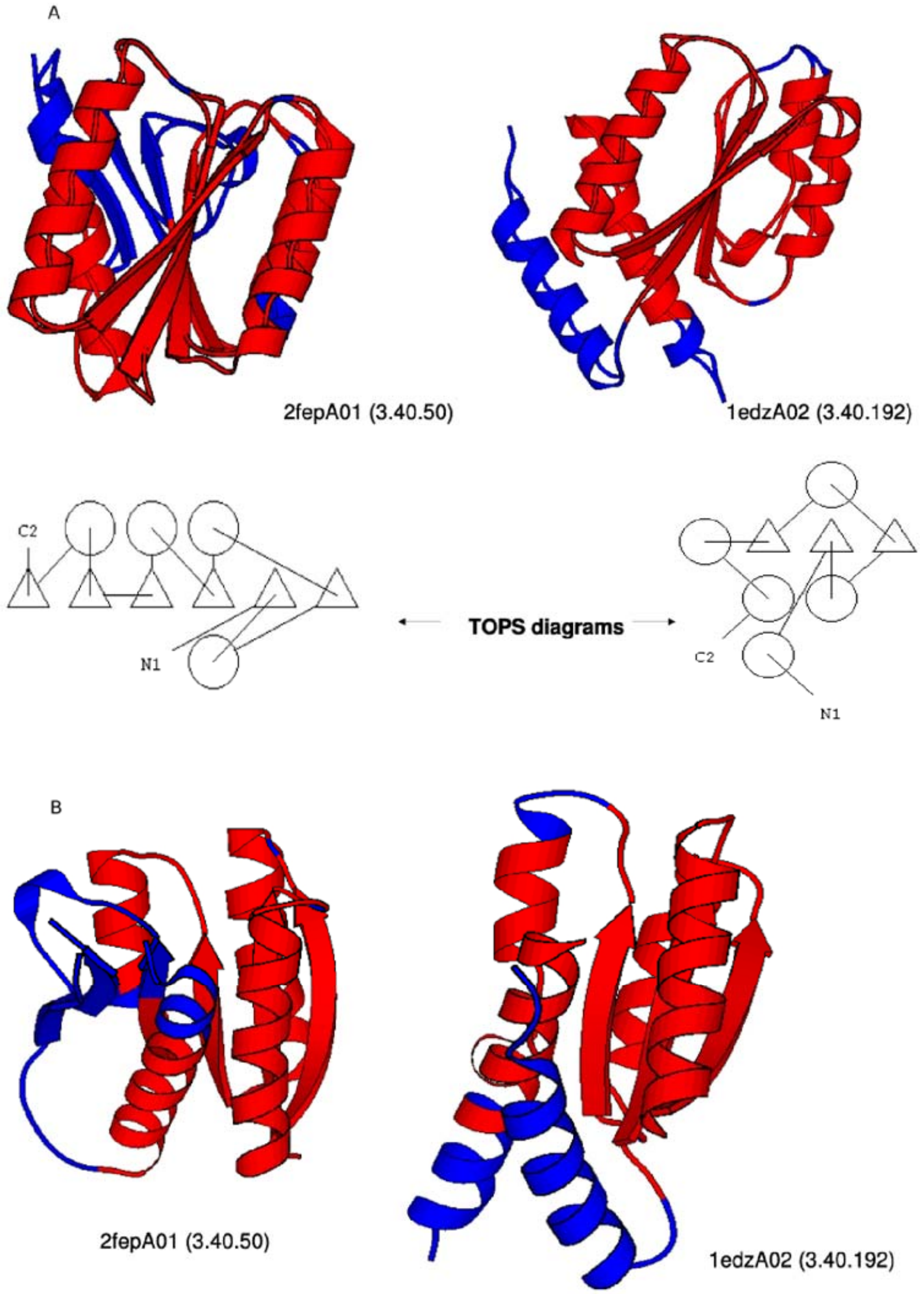

Figure S13.
Structural overlap (in red) between two domains from different αβα 3-layer folds ((a) side view, (b) front view), and the corresponding TOPS diagrams. Normalized RMSD = 3.32 Å, overlap = 60%.

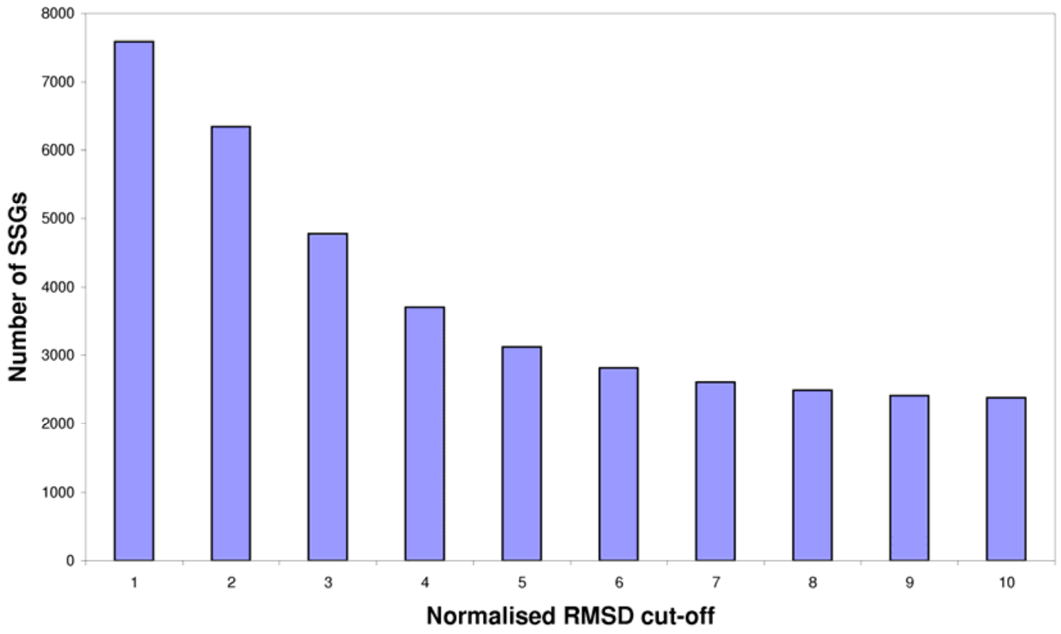

Figure S14.
Plot showing the number of SSGs identified for different normalized RMSD cut-offs.

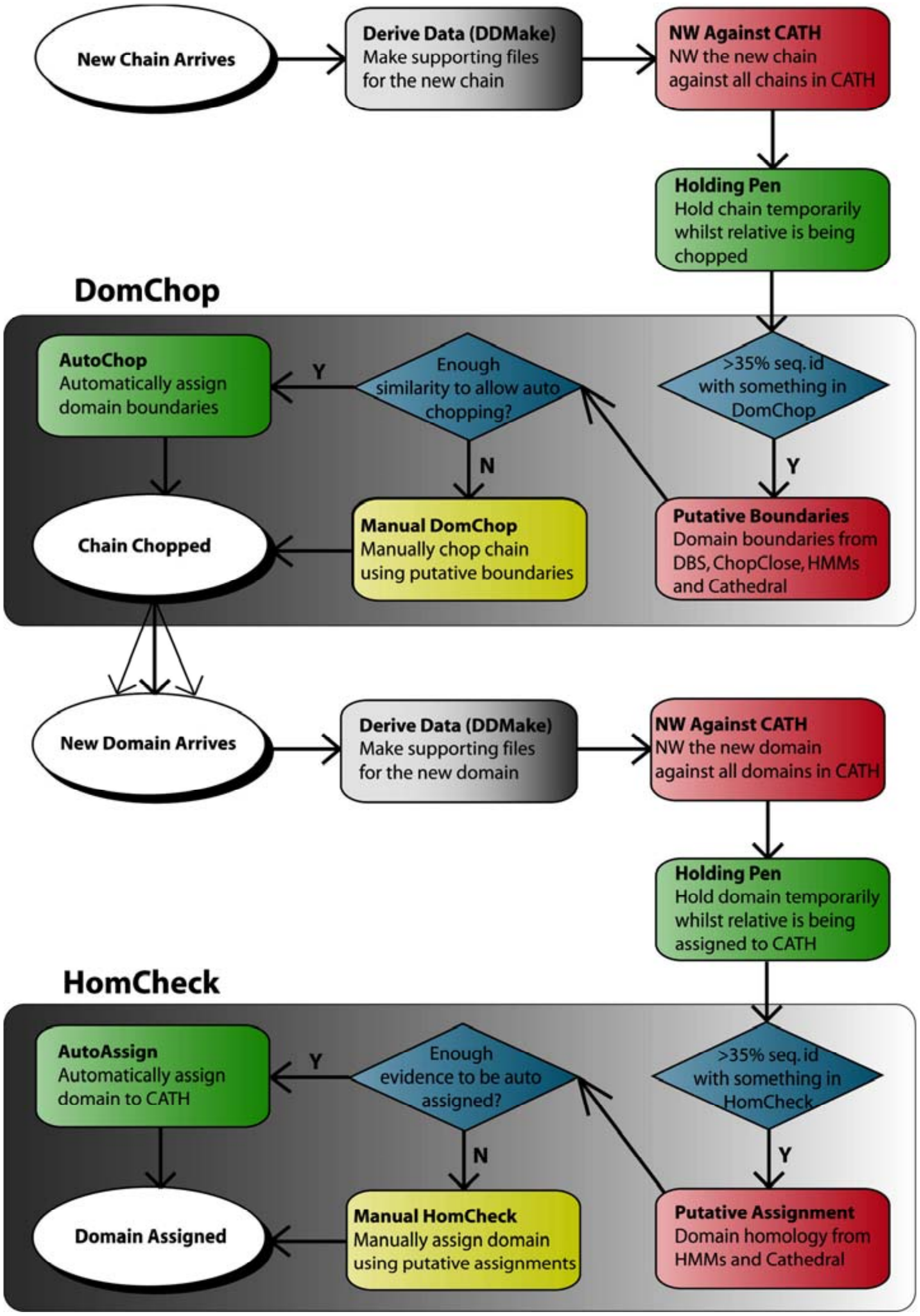

Figure S15. Schematic flow chart of the CATH update protocol
The first major step is DomChop, where one or more domains are assigned for each chain. The newly created domains are then classified into CATH superfamilies (HomCheck). As with DomChop, any domains that cannot be automatically classified are manually curated. Grey boxes denote production of metadata, blue boxes workflow decisions, yellow boxes manual curation. Definitions of abbreviations and terms used are as follows: NW (Needleman-Wunsch sequence alignment algorithm); HMM (hidden Markov model); ChopClose (assigns domain boundaroes for close homologous); CATHEDRAL (structure comparison program)

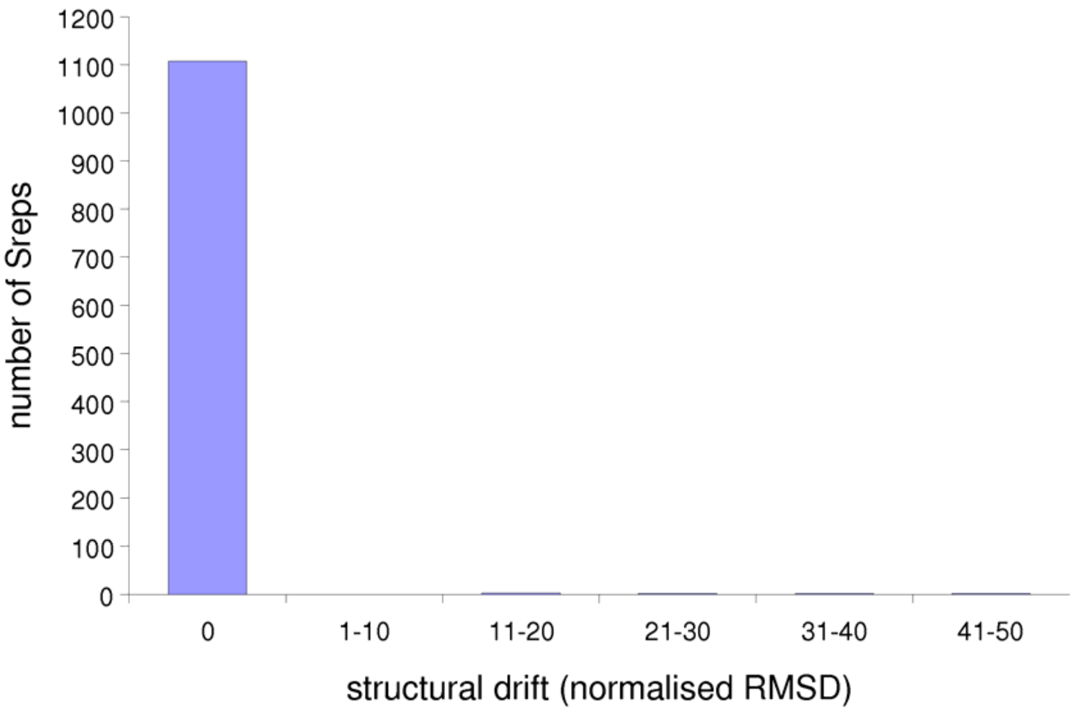

Figure S16.
Structural drift between representatives within the same S35 level (i.e., Sreps) in the CATH database (version 3.1).

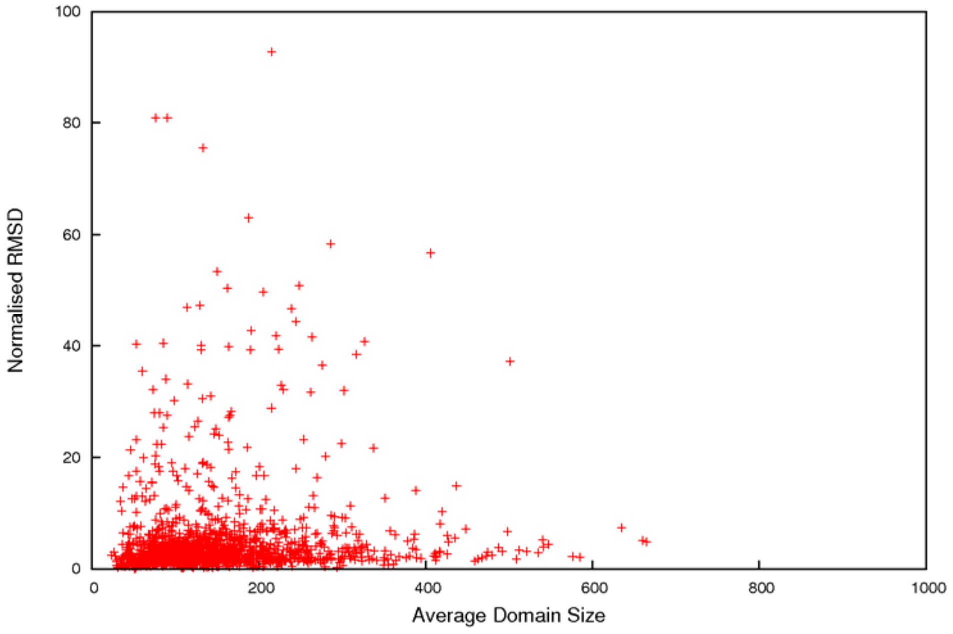

Figure S17.
Plot of normalized RMSD scores returned by pair-wise structure comparisons of homologous domains versus the average sizes of the domains being compared.

Table S1. Table showing the extent of structural diversity, structural overlap and functional diversity (measured by the number of GO terms) for each superfamily in CATH

Superfamily	# Sreps (Gene3D)	# Sreps (CATH)	Max SIMAX	# SSG's	# overlaps (T's)	# overlaps (A's)	# GO terms
1.10.10.10	6810	112	40.45	27	15	4	100
1.10.10.200	216	3	4.92	1	1	0	2
1.10.10.60	3478	50	19.86	11	14	2	66
1.10.100.10	36	3	6.24	2	0	0	9
1.10.1040.10	427	12	39.25	7	2	0	18
1.10.1060.10	266	4	4.15	1	0	0	7
1.10.110.10	65	3	5.39	2	4	1	5
1.10.1170.10	50	6	2.62	1	0	0	6
1.10.12.10	4	5	3.06	1	0	0	?
1.10.120.10	34	3	3.89	1	0	0	5
1.10.1200.10	1092	7	20.33	3	4	0	16
1.10.1200.30	1926	4	3.18	1	1	0	2
1.10.1220.10	72	5	7.48	3	1	0	2
1.10.1280.10	115	3	10.95	2	0	0	10
1.10.1300.10	68	4	2.12	1	0	0	10
1.10.132.10	3	3	1.24	1	2	1	3
1.10.150.110	30	3	2.12	1	3	2	9
1.10.150.120	58	3	2.15	1	1	0	4
1.10.150.130	674	3	3.71	1	1	0	1
1.10.150.20	520	22	40.09	8	11	2	29
1.10.150.50	161	10	7.94	2	5	1	45
1.10.150.60	40	4	5.66	2	0	0	9
1.10.150.90	2644	3	11.05	2	1	0	1
1.10.164.10	3	3	4.78	1	6	0	?
1.10.1660.10	291	4	8.43	2	7	2	1
1.10.167.10	54	3	2.24	1	2	1	10
1.10.1670.10	63	9	15.51	3	0	0	6
1.10.1680.10	424	4	5.60	2	3	1	1
1.10.196.10	16	3	1.40	1	0	0	7

Superfamily	# Sreps (Gene3D)	# Sreps (CATH)	Max SIMAX	# SSG's	# overlaps (T's)	# overlaps (A's)	# GO terms
1.10.20.10	535	20	13.13	6	4	0	23
1.10.220.10	145	12	3.50	1	9	5	14
1.10.220.20	33	3	2.63	1	4	0	8
1.10.225.10	135	4	7.04	2	7	2	7
1.10.230.10	16	4	3.09	1	0	0	3
1.10.238.10	1473	60	46.96	17	15	4	124
1.10.238.20	107	5	4.90	1	0	0	5
1.10.240.10	49	8	4.57	1	0	0	10
1.10.245.10	33	4	3.20	1	0	0	19
1.10.246.10	53	12	15.45	4	12	7	13
1.10.260.30	21	3	2.87	1	2	0	8
1.10.260.40	1676	13	12.44	4	7	3	13
1.10.268.10	12	3	2.66	1	1	1	?
1.10.275.10	52	7	22.72	3	0	0	4
1.10.30.10	238	8	5.80	2	5	2	29
1.10.300.10	6	3	1.53	1	1	0	?
1.10.3270.10	2	3	0.73	1	0	0	?
1.10.340.30	183	9	17.10	3	0	0	16
1.10.357.10	285	7	7.90	3	1	1	?
1.10.375.10	3151	5	4.95	1	0	0	2
1.10.390.10	22	3	4.03	1	1	0	?
1.10.40.30	21	9	13.00	2	6	1	2
1.10.400.10	15	3	2.22	1	0	0	3
1.10.405.10	10	3	5.41	2	0	0	?
1.10.418.10	201	13	6.07	3	1	1	46
1.10.420.10	27	6	4.26	1	0	0	2
1.10.437.10	46	8	12.38	4	0	0	11
1.10.439.10	39	3	3.54	1	0	0	?
1.10.443.10	1101	5	7.27	2	0	0	1

Superfamily	# Sreps (Gene3D)	# Sreps (CATH)	Max SIMAX	# SSG's	# overlaps (T's)	# overlaps (A's)	# GO terms
1.10.472.10	338	16	9.96	3	9	3	25
1.10.486.10	64	4	4.50	1	0	0	1
1.10.490.10	276	27	9.67	3	1	0	7
1.10.490.20	41	3	1.85	1	0	0	?
1.10.510.10	3485	28	5.68	2	0	0	207
1.10.520.10	145	7	5.05	2	0	0	5
1.10.530.10	225	9	24.17	5	0	0	6
1.10.533.10	179	15	7.72	4	2	0	54
1.10.540.10	457	8	4.97	1	1	0	8
1.10.555.10	206	3	2.83	1	0	0	46
1.10.565.10	270	17	4.28	1	0	0	44
1.10.575.10	58	3	4.06	1	0	0	2
1.10.580.10	65	4	2.81	1	0	0	3
1.10.60.10	26	3	2.03	1	1	0	?
1.10.600.10	299	9	22.46	4	0	0	15
1.10.606.10	30	3	6.71	2	0	0	?
1.10.620.20	71	7	7.15	4	0	0	4
1.10.630.10	1054	15	5.52	2	0	0	40
1.10.720.40	9	3	5.00	1	0	0	5
1.10.730.10	286	8	11.25	3	3	3	14
1.10.760.10	922	34	18.66	8	1	0	7
1.10.790.10	20	4	6.30	2	0	0	5
1.10.8.10	349	23	35.40	8	9	1	37
1.10.8.100	36	3	3.56	1	1	0	1
1.10.8.60	378	22	17.54	6	21	6	12
1.10.800.10	34	3	3.22	1	0	0	5
1.10.940.10	76	5	3.77	1	1	0	1
1.20.1050.10	465	24	12.59	2	6	2	24
1.20.1060.10	22	3	4.96	1	1	0	2

Superfamily	# Sreps (Gene3D)	# Sreps (CATH)	Max SIMAX	# SSG's	# overlaps (T's)	# overlaps (A's)	# GO terms
1.20.1070.10	1611	7	75.53	3	1	1	150
1.20.1080.10	108	4	2.42	1	0	0	21
1.20.1090.10	174	5	4.86	1	0	0	5
1.20.120.10	58	8	3.45	1	7	1	?
1.20.120.140	78	3	3.47	1	8	7	5
1.20.120.160	493	6	5.74	2	5	1	4
1.20.120.20	98	3	25.08	2	1	0	?
1.20.120.230	25	4	7.77	2	8	1	8
1.20.120.330	84	5	23.69	3	7	1	6
1.20.1250.10	137	27	26.44	9	7	1	28
1.20.1260.10	259	15	41.79	2	9	2	8
1.20.1280.50	328	3	5.05	2	0	0	15
1.20.1290.10	158	3	19.12	2	3	2	1
1.20.1300.10	65	3	6.79	2	2	0	1
1.20.1310.10	162	4	6.20	2	4	4	8
1.20.140.10	421	8	5.54	2	3	0	9
1.20.190.10	27	3	2.49	1	0	0	?
1.20.200.10	150	8	9.61	3	0	0	10
1.20.272.10	103	6	4.29	1	3	2	6
1.20.5.110	269	11	17.50	3	4	1	15
1.20.5.170	332	18	40.30	5	0	0	35
1.20.5.320	33	7	21.26	2	0	0	4
1.20.5.420	7	4	7.53	2	1	1	?
1.20.5.440	37	3	0.99	1	0	0	1
1.20.5.50	16	14	80.87	5	2	1	?
1.20.58.100	79	3	3.22	1	7	2	2
1.20.58.220	141	4	4.22	1	6	2	?
1.20.58.60	655	8	33.10	3	10	3	33
1.20.58.70	19	3	7.45	2	5	1	2

Superfamily	# Sreps (Gene3D)	# Sreps (CATH)	Max SIMAX	# SSG's	# overlaps (T's)	# overlaps (A's)	# GO terms
1.20.58.90	38	4	5.12	2	5	1	7
1.20.80.10	111	5	4.12	1	3	2	26
1.20.85.10	246	5	5.95	2	2	1	?
1.20.90.10	58	5	5.75	2	0	0	5
1.20.900.10	181	6	5.08	2	0	0	20
1.20.910.10	114	10	6.29	2	0	0	6
1.20.920.10	207	3	3.55	1	1	0	37
1.20.970.10	40	3	2.61	1	3	3	6
1.20.990.10	60	3	4.41	1	0	0	11
1.25.10.10	2883	14	56.68	12	0	0	109
1.25.40.10	6930	18	92.75	8	9	9	94
1.25.40.180	125	6	10.49	3	0	0	8
1.25.40.20	3448	18	13.15	4	0	0	92
1.25.40.80	16	4	2.97	1	1	1	2
1.25.40.90	110	5	3.59	1	1	0	19
1.50.10.10	231	13	14.82	6	0	0	11
1.50.10.100	17	5	4.95	1	0	0	?
1.50.10.20	247	9	31.94	5	0	0	31
1.50.10.50	33	3	1.78	1	0	0	7
2.100.10.10	14	3	4.94	1	0	0	?
2.100.10.30	46	3	2.91	1	0	0	7
2.102.10.10	263	8	5.83	2	0	0	11
2.110.10.10	63	5	3.17	1	0	0	16
2.120.10.10	289	9	6.22	3	0	0	2
2.120.10.30	1159	8	6.81	2	1	1	42
2.130.10.10	3740	22	10.26	8	1	1	109
2.160.10.10	483	10	6.63	3	0	0	8
2.160.20.10	788	16	14.09	7	0	0	10
2.170.130.10	178	3	3.10	1	0	0	?

Superfamily	# Sreps (Gene3D)	# Sreps (CATH)	Max SIMAX	# SSG's	# overlaps (T's)	# overlaps (A's)	# GO terms
2.170.16.10	163	5	4.39	1	0	0	10
2.170.270.10	232	3	10.64	3	0	0	39
2.170.30.10	16	3	3.98	1	0	0	?
2.30.110.10	275	9	6.18	3	0	0	2
2.30.140.10	23	4	1.60	1	2	1	2
2.30.18.10	8	4	1.83	1	1	1	7
2.30.180.10	165	3	3.10	1	0	0	13
2.30.29.30	861	38	12.69	6	1	1	121
2.30.30.100	149	11	4.26	1	1	0	10
2.30.30.110	70	4	2.65	1	0	0	1
2.30.30.160	84	7	6.36	2	0	0	37
2.30.30.30	124	7	5.38	2	5	4	11
2.30.30.40	397	43	27.93	6	6	6	109
2.30.30.50	8	4	5.89	2	2	2	3
2.30.30.90	10	3	3.89	1	0	0	?
2.30.38.10	720	4	2.20	1	0	0	18
2.30.39.10	89	15	4.45	1	0	0	9
2.30.40.10	435	12	4.53	1	0	0	2
2.30.42.10	1025	37	8.97	4	1	1	75
2.40.10.10	1831	93	80.92	14	4	3	57
2.40.10.120	138	3	2.16	1	1	1	1
2.40.100.10	183	3	1.72	1	0	0	12
2.40.110.10	308	5	3.95	1	0	0	9
2.40.128.20	202	26	17.41	5	1	1	22
2.40.128.30	9	4	2.24	1	0	0	?
2.40.160.10	294	4	4.03	1	0	0	?
2.40.160.20	582	4	4.56	1	0	0	1
2.40.170.20	1295	4	4.39	1	0	0	?
2.40.20.10	45	4	4.87	1	0	0	14

Superfamily	# Sreps (Gene3D)	# Sreps (CATH)	Max SIMAX	# SSG's	# overlaps (T's)	# overlaps (A's)	# GO terms
2.40.240.10	75	4	4.36	1	0	0	7
2.40.240.20	2	3	1.99	1	1	0	?
2.40.290.10	41	3	4.70	1	0	0	16
2.40.30.10	1621	29	22.33	6	3	1	35
2.40.30.20	198	5	5.17	2	7	2	8
2.40.33.10	55	3	1.52	1	3	1	2
2.40.340.10	12	3	3.27	1	1	0	?
2.40.37.10	58	6	6.23	2	0	0	2
2.40.40.20	205	11	5.93	2	1	0	7
2.40.50.100	877	16	27.49	5	6	5	17
2.40.50.110	14	13	6.63	2	1	1	?
2.40.50.120	42	4	4.06	1	0	0	15
2.40.50.140	1734	75	47.33	23	5	3	51
2.40.50.40	188	22	12.14	5	4	4	39
2.40.70.10	10797	30	39.82	6	0	0	18
2.60.120.10	2197	42	28.84	9	1	0	55
2.60.120.170	4	3	6.05	2	0	0	?
2.60.120.180	26	5	4.04	1	0	0	1
2.60.120.20	690	31	44.30	11	0	0	?
2.60.120.200	1243	44	46.78	14	0	0	73
2.60.120.220	3	3	4.39	1	1	0	?
2.60.120.230	15	3	12.17	3	1	0	3
2.60.120.260	745	35	12.56	8	2	0	44
2.60.120.290	437	3	2.97	1	0	0	37
2.60.120.330	184	4	7.32	2	0	0	15
2.60.120.380	172	3	3.90	1	1	0	6
2.60.120.40	64	9	3.10	1	0	0	11
2.60.130.10	33	3	4.91	1	0	0	?
2.60.20.10	84	7	2.51	1	2	0	3

Superfamily	# Sreps (Gene3D)	# Sreps (CATH)	Max SIMAX	# SSG's	# overlaps (T's)	# overlaps (A's)	# GO terms
2.60.20.30	3	4	6.93	2	1	1	?
2.60.200.10	30	4	4.25	1	0	0	18
2.60.200.20	404	8	5.42	2	0	0	38
2.60.210.10	115	3	5.78	2	0	0	11
2.60.220.10	13	6	4.90	1	1	0	?
2.60.260.20	223	6	5.78	2	0	0	9
2.60.40.10	4151	171	34.02	23	7	4	162
2.60.40.1070	59	3	3.05	1	0	0	?
2.60.40.1090	159	4	6.67	2	0	0	1
2.60.40.1180	377	24	18.76	6	7	5	15
2.60.40.150	560	9	10.46	2	0	0	66
2.60.40.200	74	4	2.80	1	1	1	7
2.60.40.290	47	3	6.18	2	0	0	?
2.60.40.30	2214	61	25.47	7	5	3	101
2.60.40.320	161	4	8.41	3	1	0	4
2.60.40.340	11	3	5.30	2	0	0	11
2.60.40.360	124	4	3.33	1	1	0	5
2.60.40.420	1270	39	15.76	6	1	0	13
2.60.40.680	21	4	3.10	1	1	0	1
2.60.40.720	19	3	6.35	2	0	0	10
2.60.40.790	307	6	4.61	1	0	0	13
2.60.40.920	179	3	1.86	1	0	0	1
2.60.60.20	80	4	4.49	1	0	0	17
2.70.130.10	88	5	3.55	1	0	0	12
2.70.40.10	86	6	4.89	1	0	0	2
2.70.98.10	143	10	7.51	3	0	0	6
2.70.98.20	16	4	2.38	1	0	0	10
2.80.10.50	390	38	6.20	3	0	0	33
3.10.100.10	488	30	8.40	4	0	0	37

Superfamily	# Sreps (Gene3D)	# Sreps (CATH)	Max SIMAX	# SSG's	# overlaps (T's)	# overlaps (A's)	# GO terms
3.10.110.10	230	9	4.31	1	0	0	22
3.10.120.10	134	7	3.97	1	0	0	20
3.10.129.10	785	19	7.36	3	0	0	18
3.10.130.10	16	6	3.27	1	0	0	12
3.10.150.10	176	6	2.86	1	0	0	1
3.10.180.10	863	20	21.42	4	0	0	9
3.10.20.10	8	8	10.52	3	3	3	7
3.10.20.120	13	7	3.17	1	0	0	1
3.10.20.240	48	5	4.65	1	1	1	15
3.10.20.30	560	22	9.89	6	2	2	18
3.10.20.90	1217	33	10.36	6	4	3	64
3.10.200.10	73	4	2.09	1	0	0	6
3.10.28.10	234	13	7.33	5	6	6	4
3.10.290.10	257	5	4.79	1	1	0	7
3.10.310.10	126	11	5.62	2	0	0	2
3.10.320.10	560	5	4.97	1	1	1	4
3.10.330.10	36	5	4.00	1	0	0	7
3.10.390.10	11	3	3.60	1	0	0	8
3.10.400.10	26	3	6.74	2	0	0	2
3.10.450.10	116	5	5.17	2	0	0	11
3.10.450.30	18	5	6.86	2	0	0	?
3.10.450.40	46	11	6.91	2	2	2	10
3.10.450.50	729	17	5.41	2	0	0	18
3.10.50.10	14	6	3.00	1	1	1	1
3.10.50.40	463	12	18.91	2	0	0	20
3.20.10.10	92	4	2.40	1	0	0	6
3.20.16.10	5	3	2.71	1	0	0	?
3.20.20.10	177	6	4.41	1	0	0	8
3.20.20.100	312	8	3.61	1	0	0	31

Superfamily	# Sreps (Gene3D)	# Sreps (CATH)	Max SIMAX	# SSG's	# overlaps (T's)	# overlaps (A's)	# GO terms
3.20.20.110	2985	3	2.95	1	0	0	3
3.20.20.120	192	12	5.39	2	0	0	6
3.20.20.140	799	19	9.12	7	0	0	23
3.20.20.150	302	7	6.07	2	0	0	2
3.20.20.190	291	6	7.40	2	0	0	13
3.20.20.20	77	3	2.99	1	0	0	3
3.20.20.210	119	3	4.36	1	0	0	4
3.20.20.220	74	3	6.20	2	0	0	5
3.20.20.240	24	3	4.83	1	0	0	1
3.20.20.30	179	6	6.31	2	0	0	?
3.20.20.40	8	3	3.07	1	0	0	?
3.20.20.60	271	11	12.66	4	0	0	9
3.20.20.70	2597	72	38.43	19	0	0	65
3.20.20.80	1278	68	37.19	17	0	0	44
3.20.70.20	81	3	7.40	3	0	0	2
3.20.80.10	134	3	3.95	1	0	0	1
3.30.10.10	6	6	4.91	1	0	0	3
3.30.110.20	27	3	1.95	1	3	1	3
3.30.110.40	75	4	6.33	2	2	0	?
3.30.1120.10	60	4	3.64	1	0	0	2
3.30.1130.10	91	4	3.27	1	1	0	3
3.30.1240.10	9	4	3.64	1	0	0	?
3.30.1310.10	50	3	1.77	1	4	1	?
3.30.1330.20	379	3	4.42	1	1	0	8
3.30.1330.30	138	8	4.91	1	2	2	12
3.30.1330.40	169	3	4.74	1	0	0	3
3.30.1360.10	106	3	3.83	1	1	0	3
3.30.1360.40	70	4	3.86	1	3	0	2
3.30.1370.10	320	9	4.53	1	6	0	26

Superfamily	# Sreps (Gene3D)	# Sreps (CATH)	Max SIMAX	# SSG's	# overlaps (T's)	# overlaps (A's)	# GO terms
3.30.1380.10	64	4	8.02	2	0	0	8
3.30.1390.10	69	3	2.39	1	2	0	5
3.30.1460.10	35	6	4.25	1	1	0	?
3.30.1460.20	20	3	4.90	1	0	0	6
3.30.1490.20	443	13	9.18	2	4	1	18
3.30.1490.70	25	6	5.97	2	1	1	1
3.30.1520.10	170	4	4.22	1	0	0	24
3.30.160.20	155	7	6.80	2	2	0	27
3.30.160.60	6541	21	16.65	3	4	1	26
3.30.190.10	32	3	2.06	1	0	0	2
3.30.200.20	1462	50	19.04	6	6	2	117
3.30.210.10	20	4	2.63	1	0	0	11
3.30.230.10	542	19	14.61	8	0	0	44
3.30.30.10	49	15	10.20	5	2	0	7
3.30.300.10	99	6	15.64	3	1	0	7
3.30.300.20	276	11	14.72	5	3	0	6
3.30.300.30	1175	4	2.31	1	2	0	19
3.30.300.50	7	3	3.54	1	3	1	?
3.30.310.10	37	4	4.75	1	3	2	14
3.30.310.50	80	3	5.02	2	3	0	4
3.30.360.10	407	19	50.46	9	2	1	14
3.30.365.10	262	13	23.92	4	1	0	5
3.30.379.10	101	4	3.71	1	0	0	4
3.30.380.10	4	3	3.34	1	0	0	?
3.30.390.10	155	13	5.34	2	0	0	5
3.30.390.30	228	11	8.15	2	1	1	14
3.30.390.50	97	4	4.45	1	1	0	5
3.30.40.10	2075	23	30.13	11	4	2	124
3.30.420.10	2100	22	28.16	9	0	0	49

Superfamily	# Sreps (Gene3D)	# Sreps (CATH)	Max SIMAX	# SSG's	# overlaps (T's)	# overlaps (A's)	# GO terms
3.30.420.130	86	3	3.36	1	2	1	?
3.30.420.140	34	3	5.73	2	1	0	?
3.30.420.40	1732	16	21.78	7	3	1	33
3.30.428.10	166	4	5.45	2	0	0	13
3.30.429.10	87	5	4.93	1	2	0	4
3.30.43.10	235	8	8.41	2	1	0	9
3.30.450.20	4020	13	11.19	3	0	0	50
3.30.450.30	42	4	3.66	1	0	0	7
3.30.450.40	1507	8	5.56	2	0	0	25
3.30.450.60	66	3	3.77	1	0	0	7
3.30.460.10	337	8	12.17	5	3	0	26
3.30.465.10	69	4	4.34	1	0	0	4
3.30.470.10	78	4	3.84	1	1	0	6
3.30.470.20	469	15	27.47	7	0	0	34
3.30.470.30	109	5	5.66	2	1	1	7
3.30.497.10	146	11	5.43	2	0	0	13
3.30.50.10	102	8	18.26	3	1	0	27
3.30.500.10	466	11	5.95	2	0	0	9
3.30.505.10	143	21	6.68	3	2	1	76
3.30.530.20	148	6	32.07	3	1	0	18
3.30.540.10	161	8	6.80	2	0	0	10
3.30.559.10	203	4	6.80	2	0	0	14
3.30.56.30	17	3	4.12	1	0	0	2
3.30.565.10	3128	15	27.15	2	0	0	61
3.30.572.10	39	4	53.44	2	0	0	2
3.30.60.10	41	4	2.04	1	0	0	4
3.30.60.20	127	5	4.23	1	2	1	42
3.30.60.30	173	10	12.54	4	6	3	20
3.30.70.100	447	12	5.63	2	12	2	24

Superfamily	# Sreps (Gene3D)	# Sreps (CATH)	Max SIMAX	# SSG's	# overlaps (T's)	# overlaps (A's)	# GO terms
3.30.70.120	116	4	4.20	1	4	1	4
3.30.70.1230	252	3	3.42	1	0	0	12
3.30.70.150	1889	4	2.49	1	0	0	3
3.30.70.20	932	19	10.74	4	1	1	13
3.30.70.240	202	3	2.93	1	7	1	8
3.30.70.260	217	4	4.88	1	3	0	4
3.30.70.270	21248	19	30.93	6	13	2	22
3.30.70.330	1318	41	32.16	10	9	3	67
3.30.70.340	42	3	3.94	1	1	0	5
3.30.70.360	94	5	3.09	1	0	0	5
3.30.70.370	91	3	9.19	2	2	1	6
3.30.70.60	57	4	3.94	1	1	0	5
3.30.70.830	27	3	1.52	1	1	0	3
3.30.70.890	63	5	5.36	2	1	0	10
3.30.70.900	276	8	5.03	2	1	0	3
3.30.70.930	21	3	2.26	1	0	0	?
3.30.70.980	7	3	2.03	1	5	0	?
3.30.700.10	479	3	4.25	1	0	0	?
3.30.710.10	511	8	4.38	1	0	0	43
3.30.750.24	378	3	3.13	1	1	1	10
3.30.830.10	528	11	5.61	3	0	0	10
3.30.870.10	402	5	6.98	3	0	0	7
3.30.9.10	77	5	7.90	2	0	0	3
3.30.930.10	553	18	50.87	7	0	0	27
3.30.950.10	89	5	5.56	2	0	0	4
3.40.1010.10	210	3	2.65	1	1	0	4
3.40.1030.10	58	3	3.35	1	0	0	6
3.40.1050.10	49	4	3.91	1	0	0	3
3.40.1080.10	51	3	6.93	2	0	0	5

Superfamily	# Sreps (Gene3D)	# Sreps (CATH)	Max SIMAX	# SSG's	# overlaps (T's)	# overlaps (A's)	# GO terms
3.40.109.10	248	4	4.71	1	0	0	?
3.40.1110.10	327	3	5.31	2	0	0	30
3.40.1160.10	175	3	4.43	1	0	0	11
3.40.1190.10	235	7	4.81	1	0	0	5
3.40.1190.20	461	14	6.27	3	0	0	13
3.40.120.10	239	7	9.21	3	1	0	7
3.40.1260.10	132	3	3.83	1	1	0	?
3.40.1280.10	244	13	5.76	2	0	0	5
3.40.1310.20	64	3	7.16	2	0	0	?
3.40.1350.10	80	5	6.50	2	1	0	4
3.40.1380.20	52	4	4.02	1	1	0	2
3.40.140.10	268	8	10.85	2	0	0	18
3.40.190.10	4138	73	42.68	15	2	0	28
3.40.190.80	90	7	4.44	1	1	0	9
3.40.192.10	172	10	18.17	3	4	1	15
3.40.20.10	177	12	8.90	2	2	1	13
3.40.220.10	157	5	6.72	2	0	0	14
3.40.225.10	82	4	4.40	1	0	0	6
3.40.228.10	70	3	2.76	1	0	0	1
3.40.250.10	623	14	10.32	5	0	0	31
3.40.30.10	2481	71	39.28	11	5	3	57
3.40.309.10	71	7	3.58	1	1	0	2
3.40.350.10	139	3	4.15	1	0	0	5
3.40.390.10	500	14	16.32	6	0	0	32
3.40.420.10	37	6	2.81	1	0	0	4
3.40.430.10	179	11	3.79	1	0	0	3
3.40.449.10	18	3	3.44	1	0	0	3
3.40.47.10	1514	14	10.67	4	0	0	26
3.40.470.10	115	4	6.50	3	0	0	6

Superfamily	# Sreps (Gene3D)	# Sreps (CATH)	Max SIMAX	# SSG's	# overlaps (T's)	# overlaps (A's)	# GO terms
3.40.50.1000	1251	21	6.12	3	0	0	57
3.40.50.10090	176	4	5.60	2	0	0	1
3.40.50.1010	271	7	9.91	4	0	0	27
3.40.50.10190	314	7	6.31	2	0	0	30
3.40.50.10420	82	3	2.96	1	0	0	3
3.40.50.10490	474	15	32.94	4	0	0	10
3.40.50.1100	328	16	13.30	4	2	0	16
3.40.50.1110	374	5	6.23	2	0	0	1
3.40.50.1220	222	13	5.25	2	0	0	28
3.40.50.1240	508	9	49.76	3	0	0	17
3.40.50.1360	90	3	7.62	2	0	0	5
3.40.50.1370	109	6	8.95	2	0	0	6
3.40.50.1380	79	5	4.49	1	0	0	7
3.40.50.1400	135	7	8.36	2	0	0	3
3.40.50.1460	77	6	7.16	2	0	0	13
3.40.50.150	4104	53	17.98	16	0	0	64
3.40.50.1580	150	9	4.00	1	0	0	11
3.40.50.170	138	3	2.74	1	0	0	8
3.40.50.1820	4182	62	31.67	16	0	0	68
3.40.50.1860	60	4	5.85	2	0	0	1
3.40.50.1950	57	4	3.27	1	0	0	7
3.40.50.1970	159	5	2.86	1	0	0	5
3.40.50.1980	706	22	14.49	10	5	0	?
3.40.50.20	357	11	11.56	5	1	0	22
3.40.50.200	371	7	3.94	1	0	0	15
3.40.50.2000	1482	14	21.63	8	0	0	35
3.40.50.2020	395	15	6.81	4	0	0	24
3.40.50.2030	16	5	6.75	2	0	0	1
3.40.50.2300	3599	41	8.75	5	7	1	30

Superfamily	# Sreps (Gene3D)	# Sreps (CATH)	Max SIMAX	# SSG's	# overlaps (T's)	# overlaps (A's)	# GO terms
3.40.50.270	106	4	4.50	1	0	0	5
3.40.50.280	74	5	4.16	1	0	0	2
3.40.50.300	18681	151	63.01	45	0	0	286
3.40.50.360	369	17	7.25	3	0	0	11
3.40.50.410	531	8	4.11	1	0	0	31
3.40.50.450	127	4	8.77	2	0	0	7
3.40.50.460	15	3	3.31	1	0	0	?
3.40.50.5600	22	3	1.99	1	0	0	2
3.40.50.620	2032	49	58.33	14	1	0	62
3.40.50.720	6463	151	39.32	25	3	2	195
3.40.50.740	177	3	4.04	1	0	0	6
3.40.50.790	42	3	2.80	1	0	0	3
3.40.50.80	394	11	4.15	1	0	0	26
3.40.50.800	182	7	2.76	1	1	0	12
3.40.50.850	158	6	4.80	1	0	0	4
3.40.50.880	624	19	20.19	4	0	0	18
3.40.50.920	229	5	4.56	1	0	0	8
3.40.50.960	24	5	2.19	1	0	0	1
3.40.50.9600	399	4	3.75	1	0	0	28
3.40.50.970	617	24	11.02	5	0	0	19
3.40.50.980	1761	8	6.68	2	0	0	21
3.40.600.10	16	4	8.02	3	0	0	1
3.40.605.10	410	8	5.09	2	0	0	22
3.40.630.10	595	17	8.86	4	0	0	42
3.40.630.30	2472	28	16.77	8	2	0	37
3.40.640.10	1273	40	6.34	4	0	0	59
3.40.710.10	692	18	6.81	3	0	0	1
3.40.718.10	112	3	3.48	1	0	0	6
3.40.720.10	474	7	6.03	3	0	0	25

Superfamily	# Sreps (Gene3D)	# Sreps (CATH)	Max SIMAX	# SSG's	# overlaps (T's)	# overlaps (A's)	# GO terms
3.40.80.10	78	3	5.33	2	0	0	8
3.40.800.10	74	4	2.83	1	0	0	4
3.40.810.20	56	3	4.46	1	0	0	6
3.40.850.10	276	4	3.94	1	0	0	20
3.40.91.10	4	3	5.75	2	0	0	?
3.40.91.20	9	3	3.79	1	0	0	?
3.40.930.10	127	3	2.65	1	0	0	1
3.40.980.10	118	6	4.43	1	0	0	5
3.50.20.10	9	3	5.23	2	0	0	?
3.50.4.10	23	4	15.72	2	1	1	1
3.50.50.60	3376	46	41.57	8	1	1	59
3.50.7.10	205	4	3.69	1	0	0	12
3.60.10.10	503	8	5.99	2	0	0	34
3.60.110.10	205	4	2.65	1	0	0	9
3.60.120.10	94	3	2.99	1	0	0	3
3.60.15.10	863	13	7.03	3	0	0	12
3.60.20.10	345	29	23.20	5	0	0	11
3.60.21.10	997	10	9.25	5	0	0	40
3.60.90.10	24	4	8.64	2	0	0	2
3.65.10.10	152	6	4.05	1	0	0	?
3.70.10.10	266	6	36.48	2	1	1	24
3.75.10.10	80	5	7.77	2	0	0	11
3.80.10.10	3797	15	40.78	7	2	1	91
3.80.20.20	85	4	4.87	1	1	0	27
3.90.10.10	338	16	18.03	4	0	0	3
3.90.1070.10	3	3	4.91	1	5	5	?
3.90.110.10	102	12	8.11	2	0	0	8
3.90.1150.10	646	43	16.31	8	1	1	23
3.90.1170.20	42	3	1.99	1	1	1	2

Superfamily	# Sreps (Gene3D)	# Sreps (CATH)	Max SIMAX	# SSG's	# overlaps (T's)	# overlaps (A's)	# GO terms
3.90.1170.30	11	3	2.76	1	1	1	4
3.90.1170.50	65	4	2.03	1	0	0	4
3.90.120.10	24	3	7.98	2	0	0	1
3.90.1240.10	7	3	3.67	1	0	0	?
3.90.1260.10	25	3	2.15	1	0	0	2
3.90.176.10	60	7	4.52	1	0	0	2
3.90.180.10	470	16	4.73	1	0	0	20
3.90.190.10	538	15	7.38	2	0	0	45
3.90.190.20	239	7	3.83	1	0	0	2
3.90.20.10	934	4	18.27	3	0	0	?
3.90.210.10	20	4	4.22	1	0	0	?
3.90.215.10	53	3	1.79	1	0	0	13
3.90.226.10	862	16	9.23	4	0	0	26
3.90.230.10	192	6	3.06	1	0	0	14
3.90.25.10	106	10	17.55	4	0	0	7
3.90.280.10	77	3	3.49	1	1	1	5
3.90.340.10	5	3	1.51	1	0	0	3
3.90.45.10	60	5	2.62	1	0	0	2
3.90.550.10	2195	21	9.29	8	0	0	63
3.90.56.10	7	4	7.85	3	0	0	?
3.90.640.10	405	5	5.41	2	1	1	10
3.90.70.10	376	15	30.47	4	0	0	24
3.90.700.10	54	4	4.84	1	0	0	4
3.90.730.10	72	6	3.14	1	0	0	3
3.90.740.10	109	4	4.07	1	0	0	6
3.90.79.10	739	14	6.57	4	0	0	26
3.90.810.10	26	4	22.31	4	0	0	15
3.90.850.10	109	4	4.57	1	0	0	4
3.90.870.10	88	5	6.68	2	0	0	4

Superfamily	# Sreps (Gene3D)	# Sreps (CATH)	Max SIMAX	# SSG's	# overlaps (T's)	# overlaps (A's)	# GO terms
3.90.930.12	110	4	3.25	1	2	2	3
3.90.950.10	97	4	5.48	2	0	0	5
